# Self‐Assembled Monolayers on Gold Surfaces of Ferrocene‐BODIPY‐Fullerene‐Helical Peptide Conjugates: Studies of Electron Transfer Processes by Ultra‐Fast Electrochemistry

**DOI:** 10.1002/psc.70142

**Published:** 2026-10-27

**Authors:** Jad Rabah, Anne Vallée, Hélène Fensterbank, Minh‐Huong Ha‐Thi, Rachel Méallet, Souhir Boujday, Emmanuel Maisonhaute, Karen Wright, Emmanuel Allard

**Affiliations:** ^1^ Université Paris‐Saclay, UVSQ, CNRS, Institut Lavoisier de Versailles Versailles France; ^2^ Laboratoire de Réactivité de Surface Sorbonne Université Paris France; ^3^ Université Paris‐Saclay, CNRS, Institut Des Sciences Moléculaires d'Orsay Orsay France; ^4^ Sorbonne Université, CNRS, Institut Parisien de Chimie Moléculaire, IPCM Paris France

## Abstract

Three complex ferrocene‐BODIPY‐fullerene‐peptide conjugates were synthesized by successive CuAAC reactions between firstly ferrocene‐BODIPY azides, then a lipoyl peptide azide, with a dual‐clickable fullerene platform. These conjugates displayed broad absorption in the visible region due to their extended π‐conjugation and the presence of an intramolecular charge transfer band from ferrocene to BODIPY, particularly in the case of BODIPY derivatives functionalized at the beta and alpha positions with ethynylFc and vinylFc groups, respectively. The terminal lipoyl peptide allowed grafting of the conjugates on gold surfaces, to form self‐assembled monolayers that were characterized by PM‐IRRAS and ultrafast electrochemical techniques. Estimation of the heterogeneous electron transfer rate constants for these monolayers revealed higher values for electron transfer from the BDP or C_60_ entities to the metal surface compared with conjugates without ferrocene units. This could be attributed to a reduced distance between the (Fc)_2_‐BODIPY‐C_60_ part and the metal surface, due to folding of the conjugates within the monolayer.

## Graphical abstract

Three ferrocene‐BODIPY‐C_60_‐peptides (T1–T3) were synthesized and grafted on gold surfaces to form self‐assembled monolayers. Calculation of the tilt angles and surface density of these SAMs using PM‐IRRAS and ultrafast electrochemical experiments revealed that less vertically oriented and densely packed monolayers were obtained compared with previously described BODIPY‐fullerene peptides.

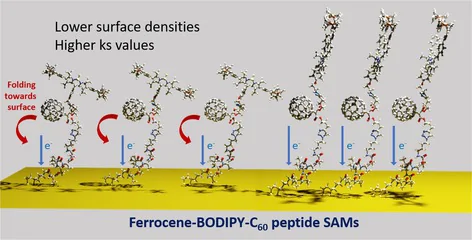

## 1 Introduction

Self‐assembled monolayers (SAMs) are formed by the interaction of molecules with a solid surface to form an ordered nanostructure. The type of solid surfaces that have been explored for monolayer formation has widened over the last 50 years, but gold remains the most extensively studied substrate, especially for electronic applications. Organic molecules can be anchored to gold surfaces via thiol functions, which have a high affinity for this metal [1, 2]. Although simple alkanethiols are often used for monolayer formation, peptides may offer certain advantages in this regard [3]. Their ability to form stable secondary structures (α‐ or 3_10_ helices, β‐sheets) through hydrogen bonding can confer a specific organization within the SAM. Additional functional groups (chromophores, redox entities) can easily be incorporated either on side chains or at the peptide terminus, along with a thiol group to allow gold attachment. For applications in the field of organic electronics, helical peptides are especially interesting. The dipole induced along a peptide helix within a SAM can facilitate electron transport processes towards the surface [4, 5]. As the helices in a SAM would have the same orientation, a cumulative macrodipole is obtained which has been shown to expedite electron transport processes [6, 7, 8, 9, 10]. Another advantage of helical peptides for SAM formation is that their relative rigidity imposes a separation of their terminal functional group, such as a chromophore, and the metal surface, which can prevent unfavorable transfer processes from the chromophore's excited state to the metal surface [11, 12]. These factors have led to investigations into the use of helical peptides for solar energy conversion applications. In SAMs where the peptide chain was substituted with different chromophores to act as electron donors/acceptors, photocurrent generation could be demonstrated upon irradiation [9, 13, 14, 15, 16, 17, 18, 19, 20].

SAMs incorporating C_60_ are also particularly interesting and have been exploited in the fabrication of solar cells as the electron selective layer [21], in field‐effect transistors [22, 23] as well as in molecular rectifiers [24]. Donor‐fullerene systems on surfaces have been used to construct devices that could generate photocurrent [25, 26, 27]. The charge separated state produced by illumination of donor‐fullerene SAMs could also function as a hole‐blocking and an electron transporting layer for photovoltaic cell applications [28].

The combination of C_60_ with helical peptides for SAM formation is therefore an attractive prospect for developing organic electronic applications. As peptide helices (α or 3_10_) have a diameter of around 1 nm [16, 29], close to that of C_60_ [30], it could be expected that organization within such a SAM could be governed both by interactions between neighboring peptide chains and between C_60_ moieties, limiting, for example, “head‐to‐tail” anomalies in the organic layer.

In a first report on C_60_‐helical peptide SAMs, Kimura et al. used a 16‐mer helical peptide ((AlaAib)_8_) to anchor C_60_ to a gold surface [17], in association with a porphyrin capped peptide, to give a heterogenous layer. Photocurrent generation could be demonstrated upon light irradiation of these mixed SAMs.

We were able to construct C_60_‐peptide conjugates suitable for SAM formation by exploiting dual “clickable” fullerene building blocks developed in our laboratory [31, 32]. In a preliminary study, a C_60_ unit was coupled to a lipoyl hexapeptide ((AibAlaAla)_2_) using a CuAAC reaction (Figure 1). The resulting assembly was able to form densely packed monolayers on a gold surface, with a tilt angle for this relatively short peptide comparable to that of the much longer peptide used in Kimura's study (37° vs. 36°). We went on to substitute this assembly with a BODIPY donor, forming a donor‐acceptor dyad system with the C_60_ unit (Figure 1) [33]. Again, the BODIPY‐C_60_‐peptides were able to form relatively dense monolayers. Ultra‐fast electrochemistry and photocurrent generation studies were carried out on these monolayers, which highlighted the important role played by the peptide anchor—analogous assemblies where the peptide was replaced by an alkyl chain gave a weaker photocurrent response.

**FIGURE 1:**
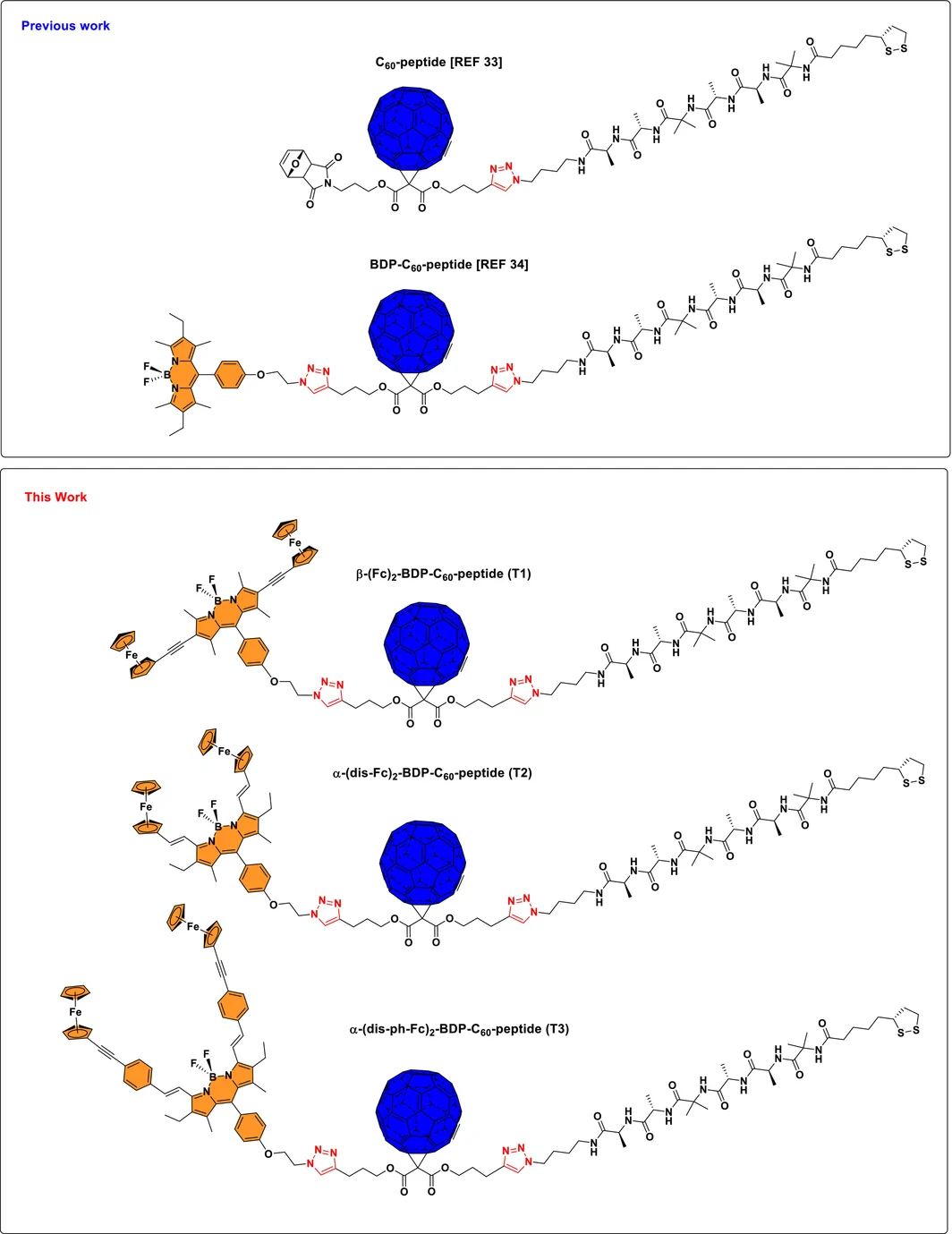
Chemical structures of previously described C_60_‐peptide and BDP‐C_60_‐peptide, and target molecules (Fc)_2_‐BDP‐C_60_‐peptides presented in this work.

To widen the scope of the BODIPY‐C_60_ dyads, we substituted the BODIPY core with additional redox active ferrocene units (Figure 1) [34]. It was expected that these modifications would result in efficient light harvesting systems with a broader absorption range than the parent compounds. It was also hoped that these tetrads would display improved charge‐separated state lifetimes, an important factor for solar energy conversion [35, 36]. In this paper, we report the synthesis and characterization of assemblies made from these tetrads and the previously used lipoyl hexapeptide. These assemblies were used for SAM formation on gold electrodes, and their electrochemical properties studied.

## 2 Results and Discussion

### 2.1 Synthesis and Characterization of Target and Reference Molecules

#### 2.1.1 Synthesis

The chemical structures of the three target molecules (Fc)_2_‐BDP‐C_60_‐peptides **T1**, **T2**, and **T3** are depicted in Figure 1. The methanofullerene unit, which acts as an electron acceptor, is functionalized on one side by the helical peptide and on the other side by the (Fc)_2_‐BDP, which possesses light‐harvesting and electron donor properties. For the (Fc)_2_‐BDP conjugates, the two Fc entities are linked to the β‐ or α‐ positions of the BODIPY core via a conjugated spacer to promote good electronic communication between the redox centers. Finally, the (Fc)_2_‐BDP and peptide are linked to the fullerene fragment via triazole and alkyl linkers. These sophisticated assemblies were prepared using our previously reported strategies on the post‐functionalization of fullerene derivatives by click chemistry processes [31, 33]. The synthetic procedure for the preparation of the precursors and target derivatives is presented in Schemes 1, 2, 3. We firstly prepared the azido ferrocene‐BODIPY derivatives **2a‐c** with moderate to good yields (47%–77%) using a one‐pot procedure starting with ferrocene‐BODIPY derivatives **1a‐c** (Scheme 1) [34].

**SCHEME 1:**
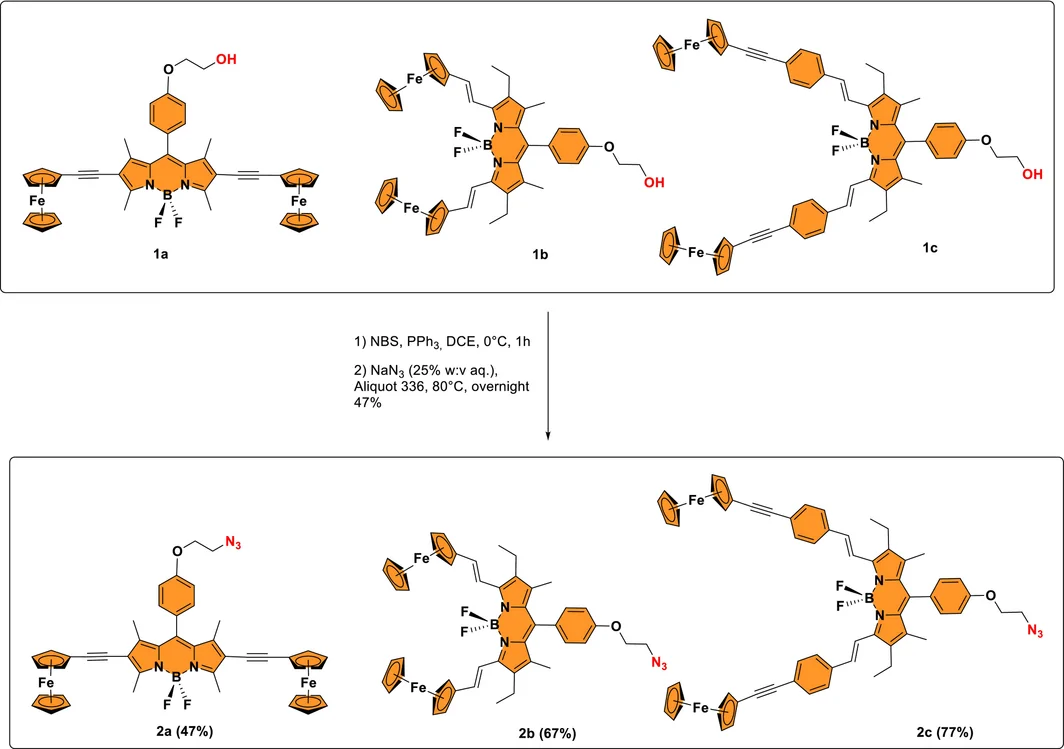
Synthesis of (Fc)_2_‐BDP azide derivatives **2a**‐**c**.

**SCHEME 2:**
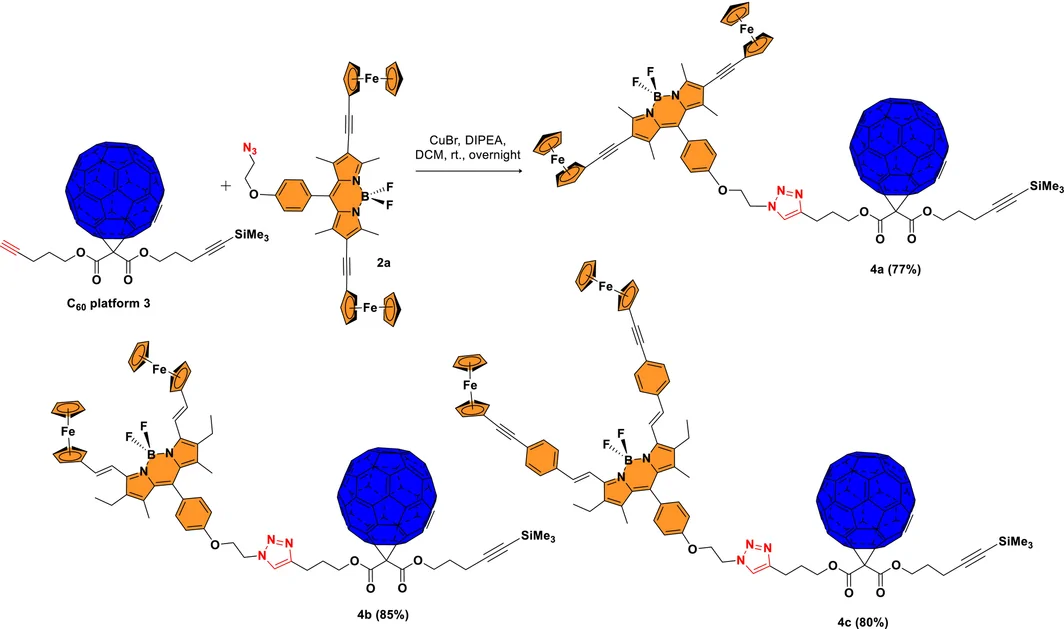
Synthesis of (Fc)_2_‐BDP‐C_60_ tetrads **4a‐4c**.

**SCHEME 3:**
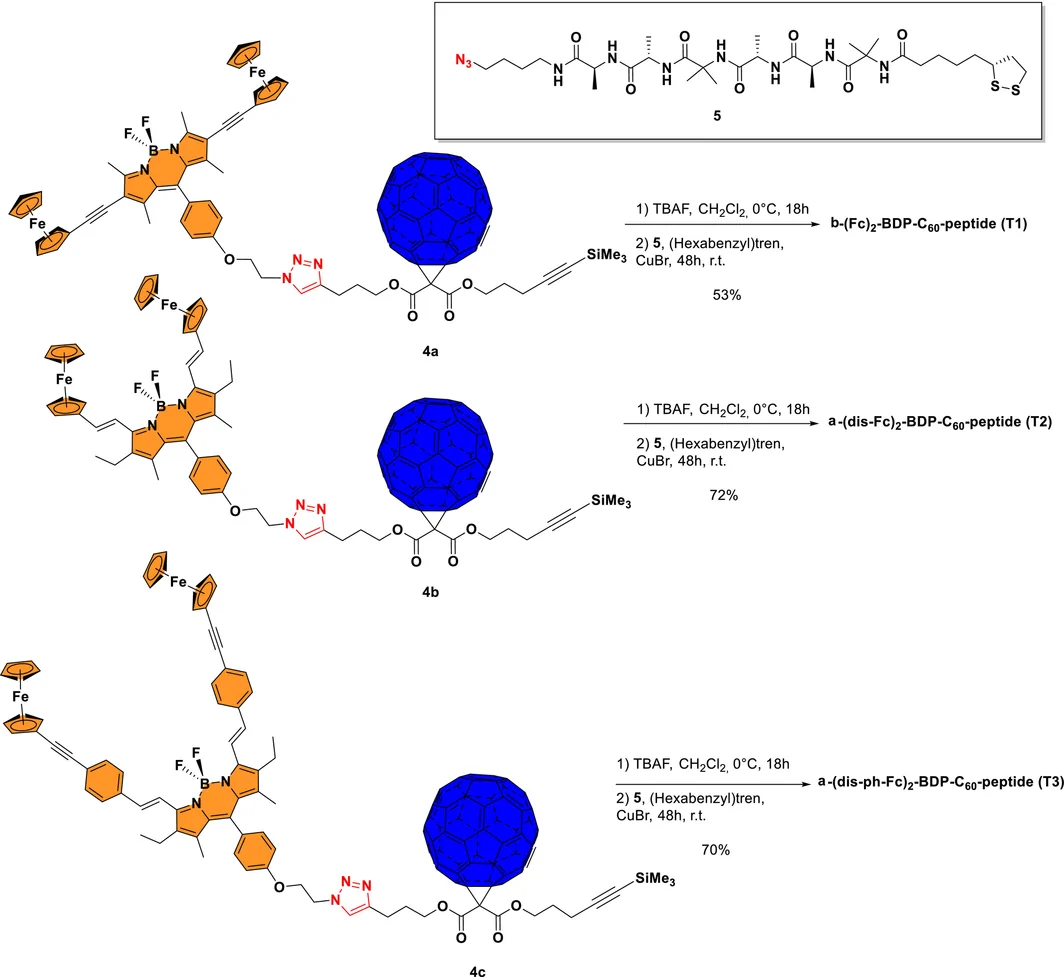
Synthesis of target molecules **T1**, **T2**, and **T3**.

These azido (Fc)_2_‐BDP derivatives were then grafted onto the clickable methanofullerene C_60_ platform **3**, which carries an alkyne on one side and a protected alkyne on the other, via a copper‐catalyzed alkyne‐azide cycloaddition (CuAAC) reaction [34]. Briefly, the reaction of the azido (Fc)_2_‐BDP derivative **2a** with the platform **3** in the presence of CuBr and DIPEA under mild conditions yielded the target tetrad (Fc)_2_‐BDP‐C_60_ **4a** in 77% yield after facile purification by column chromatography. Similarly, the reaction of **2b** and **2c** with the platform **3** under the same conditions afforded the target (Fc)_2_‐BDP‐C_60_ tetrads **4b** and **4c** in 85% and 80% yields, respectively (Scheme 2).

Finally, the target assemblies **T1**, **T2**, and **T3** were obtained in a subsequent two‐step approach (Scheme 3), developed recently in our group [31, 33]. This strategy consists in the deprotection of the TMS alkyne at first of the tetrad, followed by a click chemistry CuAAC reaction in situ with a hexapeptide. Therefore, these assemblies **T1**, **T2**, and **T3** were obtained by subjecting the corresponding parent tetrads **4a‐4c** to deprotection using TBAF in DCM, followed by a click reaction with the hexapeptide‐azide **5** using copper(I) bromide CuBr and (hexabenzyl)tren [37] as a bulky ligand in dichloromethane for 48 h. Despite the structural complexity of this family of molecules, all these derivatives were isolated in moderate to good yields (53%–72%) after purification by column chromatography.

#### 2.1.2 Characterization

All the new compounds were characterized by NMR spectroscopy and mass spectrometry. The corresponding data, in addition to the complete assignment of the signals, are provided in the experimental section (see Supporting Information). As examples, the ^1^H NMR spectra in deuterated pyridine C_5_D_5_N of the target assemblies **T2** and **T3** with the complete proton assignments are given in Figures 2 and 3. These spectra show characteristic signals of all the components of the assemblies (ferrocene, BODIPY, alkyl, and peptide chains). Furthermore, the typical singlet of the triazole unit (denoted Hm) is clearly observed, which confirms the grafting of the peptide on the tetrad. In addition, the amide proton resonances of the peptide chain display the same characteristics as those described in previous studies [32, 33], which confirms once again that the helical conformation of the peptide is retained in these target assemblies and is not altered by the presence of the tetrad.

**FIGURE 2:**
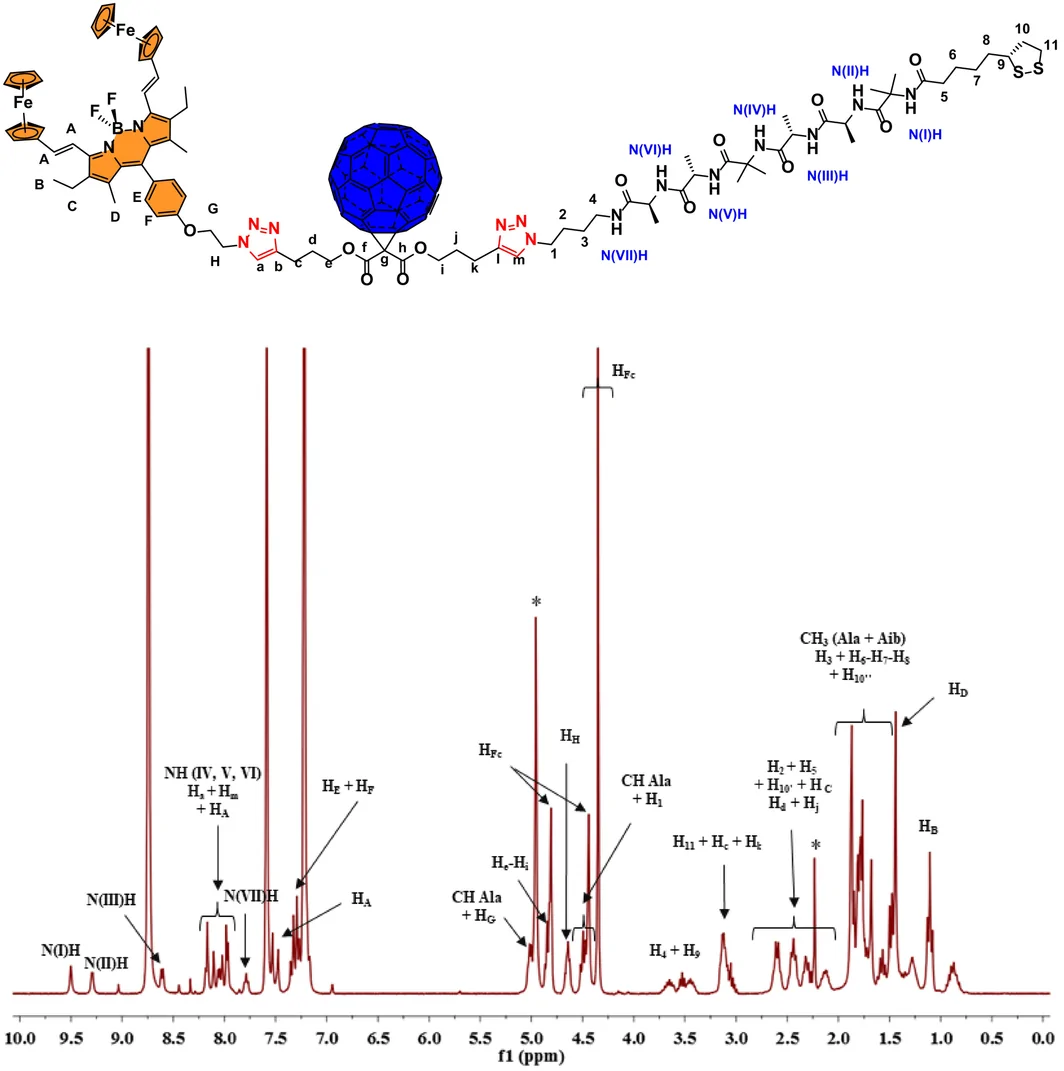
^1^H NMR spectra in C_5_D_5_N (300 MHz, 298 K) of the target assembly **T2**. The peaks labelled with an asterisk are due to water residues and acetone.

**FIGURE 3:**
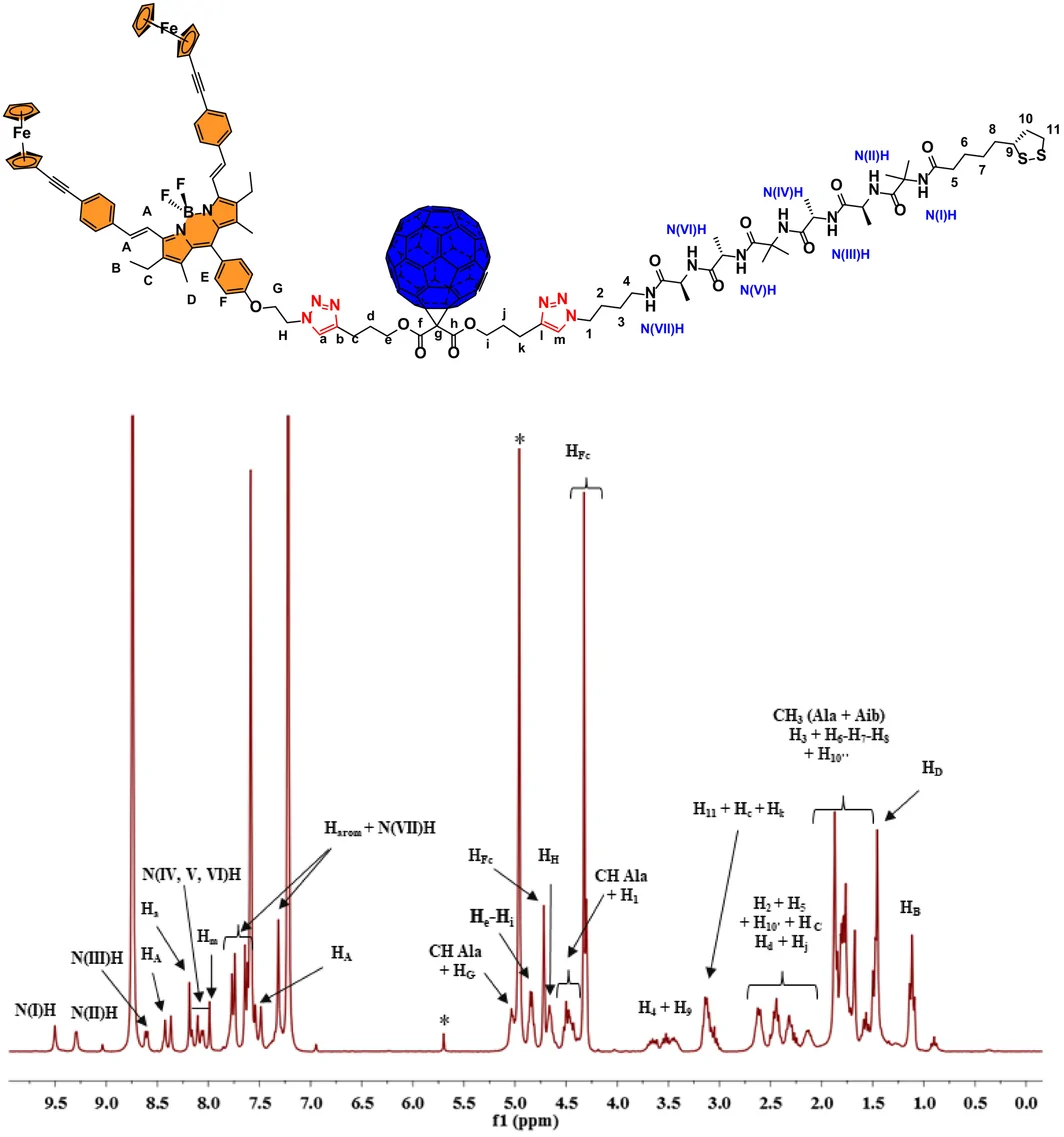
^1^H NMR spectra in C_5_D_5_N (300 MHz, 298 K) of the target assembly **T3**. The peaks labelled with an asterisk are due to water and DCM residues.

### 2.2 Photophysical Studies

Absorption spectra of triad (Fc)_2_‐BDP precursors (**1a**‐**1c**) and (Fc)_2_‐BDP‐C_60_ tetrads (**4a**‐**4c**) recorded in DCM are shown in Figure 4. The collected data are summarized in Table S1. It should be noted that these measurements were also performed on the (Fc)_2_‐BDP‐C_60_‐peptide derivatives **T1**‐**T3** (see Figure S45) as controls, in order to demonstrate the negligible contribution of the hexapeptide to the photophysical properties, as had already been observed. To demonstrate the contribution of the ferrocene unit in these systems, the precursors **1a‐c** were compared with model compounds (**1d**, **1e**, and **DSBDP**) where ferrocene was replaced by a phenyl group (see Figures S41–S43). As previously described for the β‐extended ferrocene BODIPY derivatives [34], compound **1a** exhibits strong absorption in the visible region, between 400 and 700 nm, with a bathochromic shift of the main transition band compared with a typical BODIPY, the same bathochromic shift being observed for the model compound **1d** (see Figure S41). This is attributed to the extended π‐conjugation of the BDP core by the two ethynylFc (**1a**) or the two phenylethynyl units (**1d**). In addition, the broader absorption band observed, compared with the typical absorption of BODIPY and **1d**, is also due to the presence of an intramolecular charge transfer (ICT) band from Fc to BDP which overlaps the S_0_–S_1_ transition. Regarding the α‐extended BODIPY derivatives, compound **1b** also exhibits very broad and intense absorption in the visible range compared with **DSBDP** (see Figure S42), extending from 465 to 850 nm. The width of this band is assigned, as with **1a**, to the additional ICT signature (around 690 nm) which overlaps the main transition band of BODIPY. Finally, compound **1c** exhibits strong absorption in the visible range, between 500 and 700 nm, with a more pronounced bathochromic shift of the S_0_–S_1_ BDP transition than those observed for **1a** and **1b**. This is attributed to greater conjugation of the resulting BODIPY π‐system, induced by the additional styryl and phenylalkyne subunits, the same feature being observed for the model compound **1e** (see Figure S43). It is worth noting that the absorption bands of **1c** are not as broad as in the case of β‐ethynylFc BDP **1a** and α‐divinylFc BDP **1b** (presence of additional ICT band); its absorption overlaps those of **1e**. This finding suggests a weaker electronic interaction between the Fc donors and the BDP acceptor, brought about by a more extended π‐system.

**FIGURE 4:**
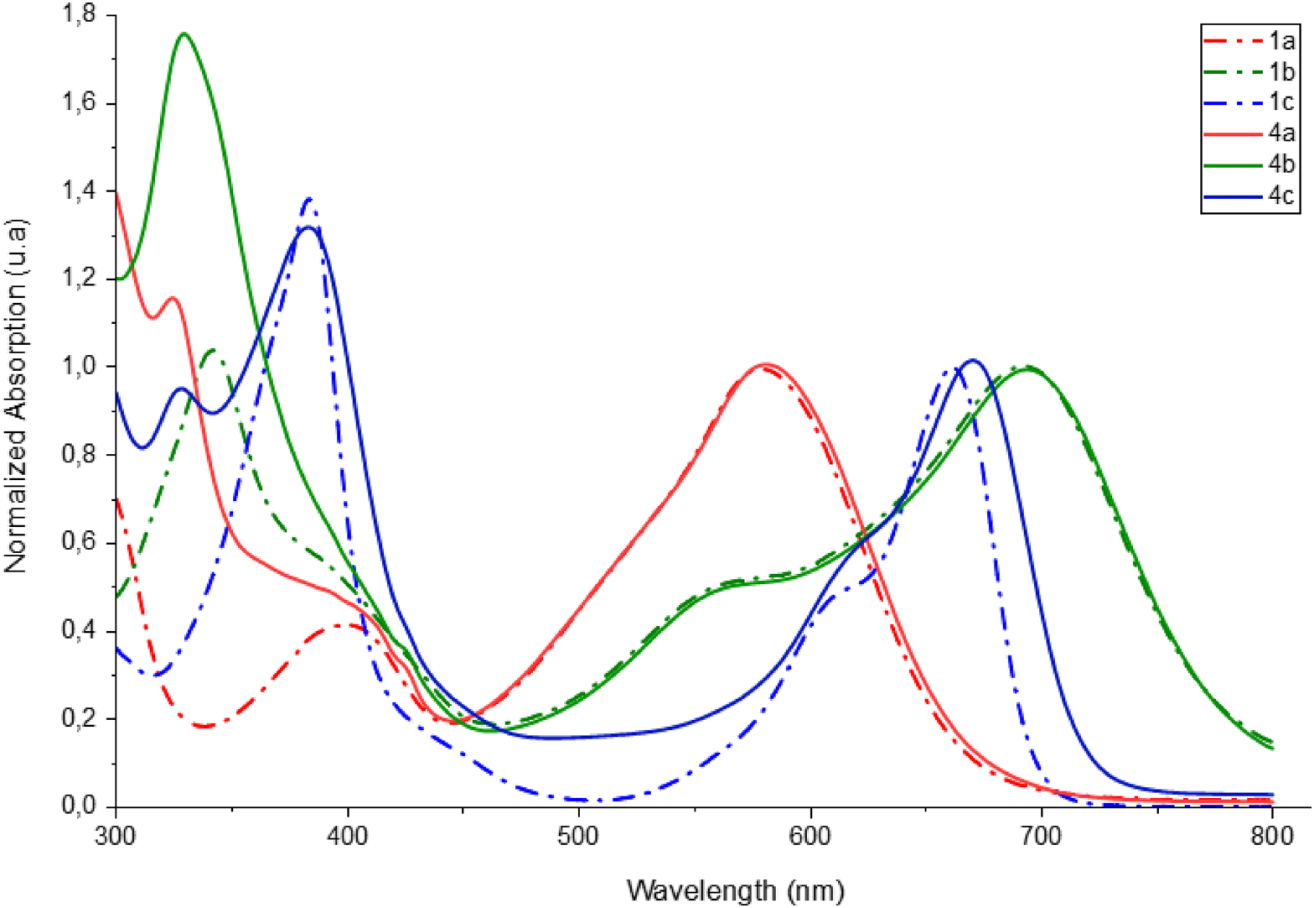
Normalized absorption spectra of (Fc)_2_‐BDP‐C_60_ target assemblies **4a**‐**4c** and their corresponding (Fc)_2_‐BDP precursors **1a**‐**1c**.

The absorptions of the (Fc)_2_‐BDP‐C_60_ tetrads (**4a**‐**4c**) are the sum of the absorption of fullerene platform **3** and the (Fc)_2_‐BDP precursors (**1a**‐**1b**). In the visible and NIR region, the intense absorption bands of (Fc)_2_‐BDP triads and (Fc)_2_‐BDP‐C_60_ tetrads overlap perfectly; these bands are attributed to the BDP centered S_0_ → S_1_ transition and ICT signature bands from Fc to BDP (for **1a**/**4a** and **1b**/**4b**). The additional absorption bands in the UV region of the spectrum, around 260 and 330 nm, are clearly attributed to the fullerene‐centered transition (see Figures S44 and S45). These findings suggest a negligible ground state interaction between Fc‐BDP and the fullerene moieties within the tetrads.

Steady‐state fluorescence analyses were performed on the triad (**1a**‐**1c**) and tetrad (**4a**‐**4b**) in DCM. The recorded emission spectra of **4b**‐**4c** are provided in Figure S46 with the corresponding emission data of all assemblies, listed in Table S1. Note that the fluorescence properties of **1a**‐**4a** have already been studied in detail [34]. All the (Fc)_2_‐BDP triads and (Fc)_2_‐BDP‐C_60_ tetrads showed a very strong quenching of fluorescence. In addition, the calculated quantum yield values of the tetrads (**4a**‐**4b**) were significantly lower than those of the triads (**1a**‐**1b**). This behavior can be rationalized in terms of photoinduced electron‐transfer processes involving the Fc and C_60_ units and the photoexcited BDP chromophore. As demonstrated in detail for the compounds **1a** and **4a** in our previous study [34], excitation of the BDP unit can trigger different electron‐transfer pathways depending on the molecular conformation. Photoinduced electron transfer can occur either from the electron‐donating Fc unit to the excited BDP (ΔG = −0.61 eV) or from the excited BDP to the electron‐accepting C_60_ unit (ΔG = −0.38 eV). In the latter case, the initially formed charge‐separated state undergoes further charge rearrangement, leading to the Fc^+^–BDP–C_60_^−^ charge‐separated state. Both primary electron‐transfer processes occur rapidly and efficiently compete with the radiative decay of the BDP excited state, resulting in its shortened lifetime and the strong fluorescence quenching. Although the position of the Fc substituent on the BDP core differs among the investigated compounds, which may influence the electronic coupling and electron‐transfer kinetics between the BDP and Fc units, efficient photoinduced electron transfer is expected to remain the dominant deactivation pathway in **1b**/**4b** and **1c**/**4c**, consistent with their strongly quenched fluorescence. The peptide moiety is electronically decoupled from the photoactive framework and is therefore not expected to introduce an additional electron‐transfer pathway.

### 2.3 Electrochemical Studies in Solution

The electrochemical studies by cyclic voltammetry (CV) of (Fc)_2_‐BDP‐C_60_ tetrads (**4a**‐**4c**) and triad precursors (**1a**‐**1c**) were first conducted in solution before the immobilization and characterization of the target assemblies (**T1**‐**T3**) on gold surfaces. All experiments were performed in a standard three‐compartment electrochemical cell under argon atmosphere at room temperature in anhydrous dichloromethane solutions, using tetra‐*n*‐butylammonium hexafluorophosphate (TBAPF_6_) as supporting electrolyte, and ferrocene (Fc) as internal reference. The recorded cyclic voltammograms are displayed in Figures 5 and S47. The half‐wave potentials (V vs Fc^+^/Fc) of the precursors and target compounds are collected in Table 1. The recorded cyclic voltammograms of the (Fc)_2_‐BDP‐C_60_ target assemblies (**4a**‐**4c**) represent the superposition of the redox signatures corresponding to the (Fc)_2_‐BDP precursors (**1a**‐**1c**) and the fullerene platform **3**.

**FIGURE 5:**
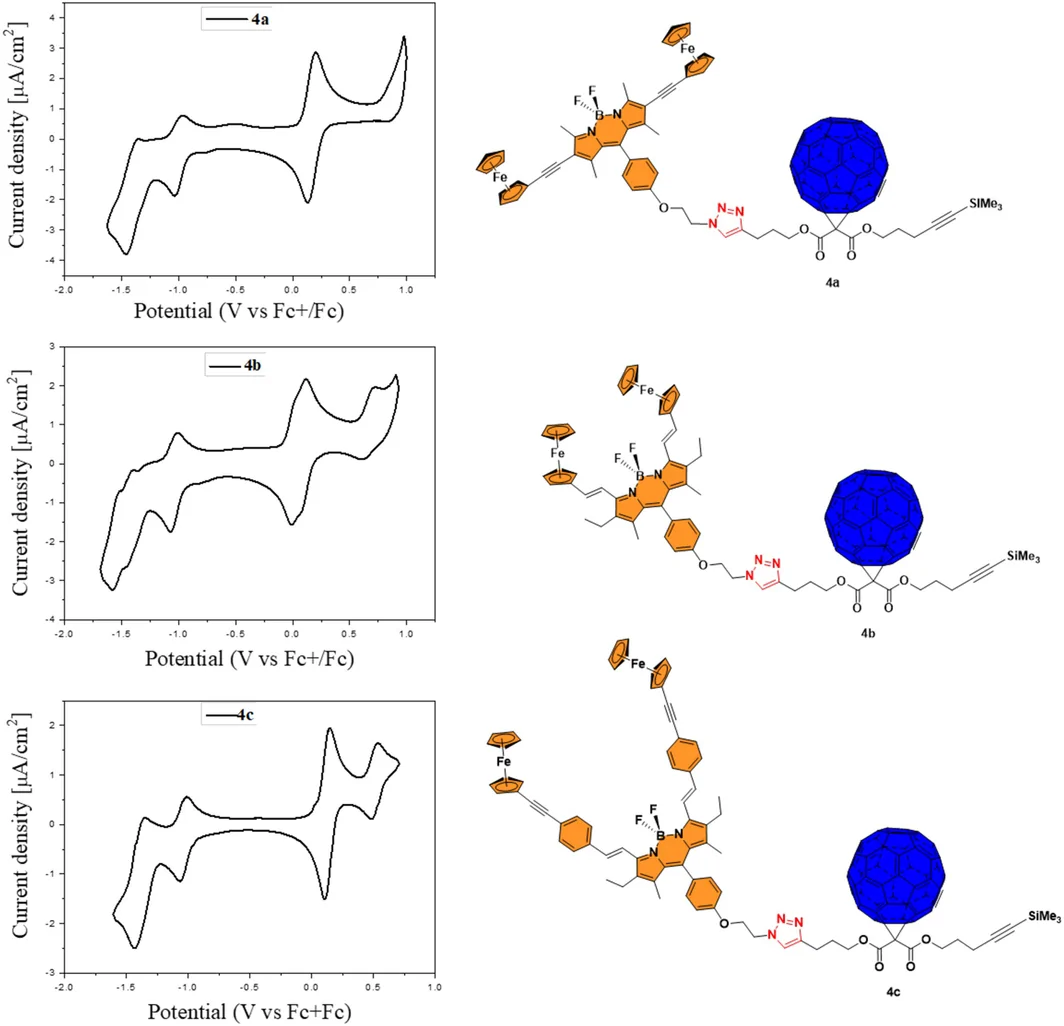
Cyclic voltammograms of the tetrads **4a‐4c** in anhydrous and oxygen‐free DCM, 0.1 M of *n*‐Bu_4_NPF_6_, pseudo‐reference electrode: Ag wire, working electrode: Pt, auxiliary electrode: Pt, scan rate: 100 mV·s^−1^.

**TABLE 1:** Electrochemical half‐wave potentials (V vs. Fc^+^/Fc) of the target assemblies **4a**‐**4c** and their precursors **1a**‐**1c**.

Compound	Potential (V vs. Fc^+^/Fc)				
	BDP^0/+^	Fc^0/+^	C_60_^0/−^	C_60_^−/2−^	BDP^0/−^
**1a**	+0.88	+0.08	—	—	−1.55
**1b**	+0.66	+0.0 +0.08	—	—	−1.54
**1c**	+0.49	0.10	—	—	−1.38
**4a**	‐^a^	+0.14	−1.01	−1.43	−1.43
**4b**	+0.66	+0.0 +0.08	−1.04	−1.44	−1.54
**4c**	+0.51	0.13	−1.03	−1.38	−1.38

^a^ Wave not well defined.

The electrochemical properties of the β‐substituted (Fc)_2_‐BDP‐C_60_ **4a** and precursor **1a** have already been described [34]. Briefly, the recorded CV of tetrad **4a** displayed in the anodic region the reversible two‐electron oxidation of the Fc units into (Fc^+^)_2_ at 0.14 V (vs. Fc^+^/Fc), whereas the oxidation of the BDP entity into the BDP^+•^ radical cation occurred at more oxidative potentials. Although partially reversible signals could be obtained for **4b** and **4c** (see below), **4a** displayed a chemically irreversible wave evidencing a decomposition reaction. In the cathodic region, two quasi‐reversible reduction waves appeared at −1.01 and −1.43 V (vs. Fc^+^/Fc). The first wave is attributed to the formation of the fullerene radical anion C_60_^•‐^, whereas the second signature is assigned to two overlapping reductions corresponding to the dianion C_60_^2‐^ and BDP^•‐^ species.

The electrochemical analyses on the α‐substituted (Fc)_2_‐BDP (**1b**‐**1c**) and (Fc)_2_‐BDP‐C_60_ (**4b‐4c**) assemblies are shown in Figures 5 and S47. Starting with the α‐bis‐vinylFc‐BDP‐C_60_ assembly **4b** and its precursor **1b**, their recorded CV exhibited in the anodic region two sequential reversible one‐electron waves at 0.00 and 0.08 V (vs. Fc^+^/Fc) corresponding to the oxidation of each Fc unit. The half‐way potential split by 80 mV is likely due to electrostatic repulsion between Fc centers in **4b** in comparison with **4a** and **4c**, rendering the oxidation of the second Fc donor harder once the first is oxidized [38]. At higher potentials, a reversible one‐electron oxidation wave appeared at 0.66 V (vs. Fc^+^/Fc) that corresponds to the formation of the BDP^+•^ radical cation species. In the cathodic region, the CV of **4b** shows a first one‐electron reduction wave at −1.04 V (vs. Fc^+^/Fc) associated with the formation of C_60_^•‐^ and a second irreversible signature attributed to the formation of the dianion C_60_^2‐^ and BDP^•‐^ species. Finally, the CV of the α‐bis‐styrylFc‐BDP‐C_60_ assembly **4c** and its precursor **1c** displays in the anodic region a reversible oxidation wave at 0.13 V (vs. Fc^+^/Fc) that corresponds to the formation of (Fc^+^)_2_. In addition, a reversible one‐electron oxidation wave, less anodically shifted (0.51 V vs. Fc^+^/Fc) in comparison to **1b**‐**4b,** is observed and assigned to the formation of BDP^+•^. In the cathodic region, the CV of **4c** shows two well‐defined reduction waves at −1.03 and −1.38 V (vs. Fc^+^/Fc). The former signature is attributed to the formation of C_60_^•‐^ and the latter reduction is associated with the formation of the dianion C_60_^2‐^ and BDP^•‐^ species.

### 2.4 SAM Formation and Characterization

All the target assemblies were individually immobilized on gold surfaces by exposing a clean gold plate to a 10^−4^ M oxygen‐free solution of **T1**, **T2**, or **T3** in dichloromethane in the dark for 18 h at room temperature under argon atmosphere. The SAMs were then rinsed with DCM and water and finally dried with a stream of nitrogen (Figure 6). The resulting SAMs were characterized by surface analysis and CV in order to gain insight on their nanoscale organization.

**FIGURE 6:**
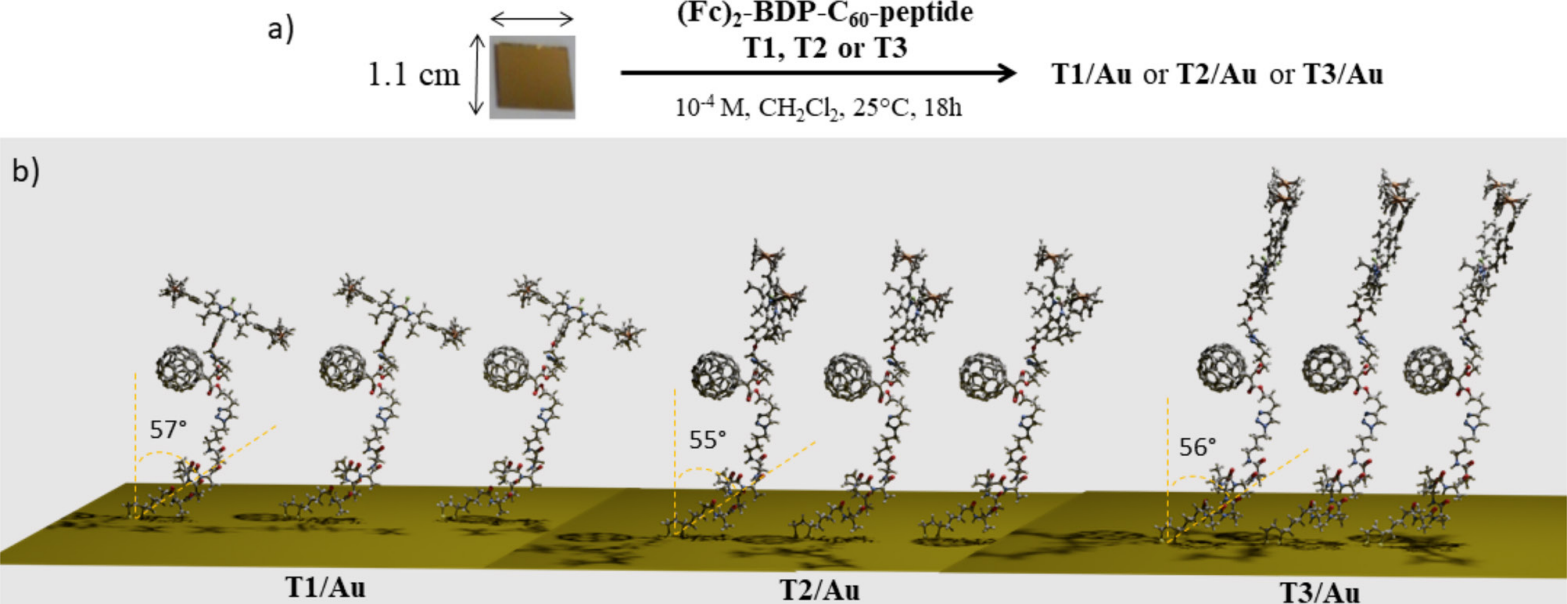
(a) General procedure for the preparation of SAMs on gold surfaces; (b) Schematic representation of SAMs of the target assemblies **T1**, **T2**, and **T3** in an ideal extended conformation along with the tilt angles of the helices from the surface normal.

#### 2.4.1 Surface Analyses

The SAMs formed by the target molecules **T1**, **T2**, and **T3** were studied at first by PM‐IRRAS in order to get insights on their conformation and orientation on gold surfaces. Figure 7 shows representative IRRAS spectra of the SAMs formed by these assemblies. Firstly, amide I and amide II bands appeared around 1660 and 1541 cm^−1^, respectively, in all the peptide assemblies (see Table 2), consistent with the retention of a helical conformation in the SAMs. These results are in accordance with those obtained in our previous studies [33], and with helical peptide‐based SAMs reported in the literature [18, 39]. Additional bands that are characteristic of the functional groups in the target molecules are also observed in the PM‐IRRAS SAMs spectra. For example, the C=O malonate characteristic band of the methanofullerene is observed around 1740 cm^−1^. The average orientation of the peptide helices was estimated from the amide I/amide II absorbance ratios. The experimental ratios were 1.48, 1.63, and 1.56 for **T1**, **T2**, and **T3**, respectively. Assuming an α‐helical geometry, with transition‐moment angles of 39° and 75° for the amide I and amide II modes, respectively, and using a scaling factor (K = 1.67), effective tilt angles of approximately 57.5°, 55.1°, and 56.2° were obtained. Because these values lie close to the magic angle (54.7°), they should not be interpreted as evidence for a narrowly defined preferential orientation. Rather, they correspond to effective orientational averages and are also compatible with a relatively broad distribution of helix orientations. Recalculation using a 3_10_‐helical geometry (45° and 90° for the amide I and amide II transition moments, respectively) changes the calculated tilt angles by less than approximately 0.3°, showing that the assumed helix type has only a minor effect in the present case.

**FIGURE 7:**
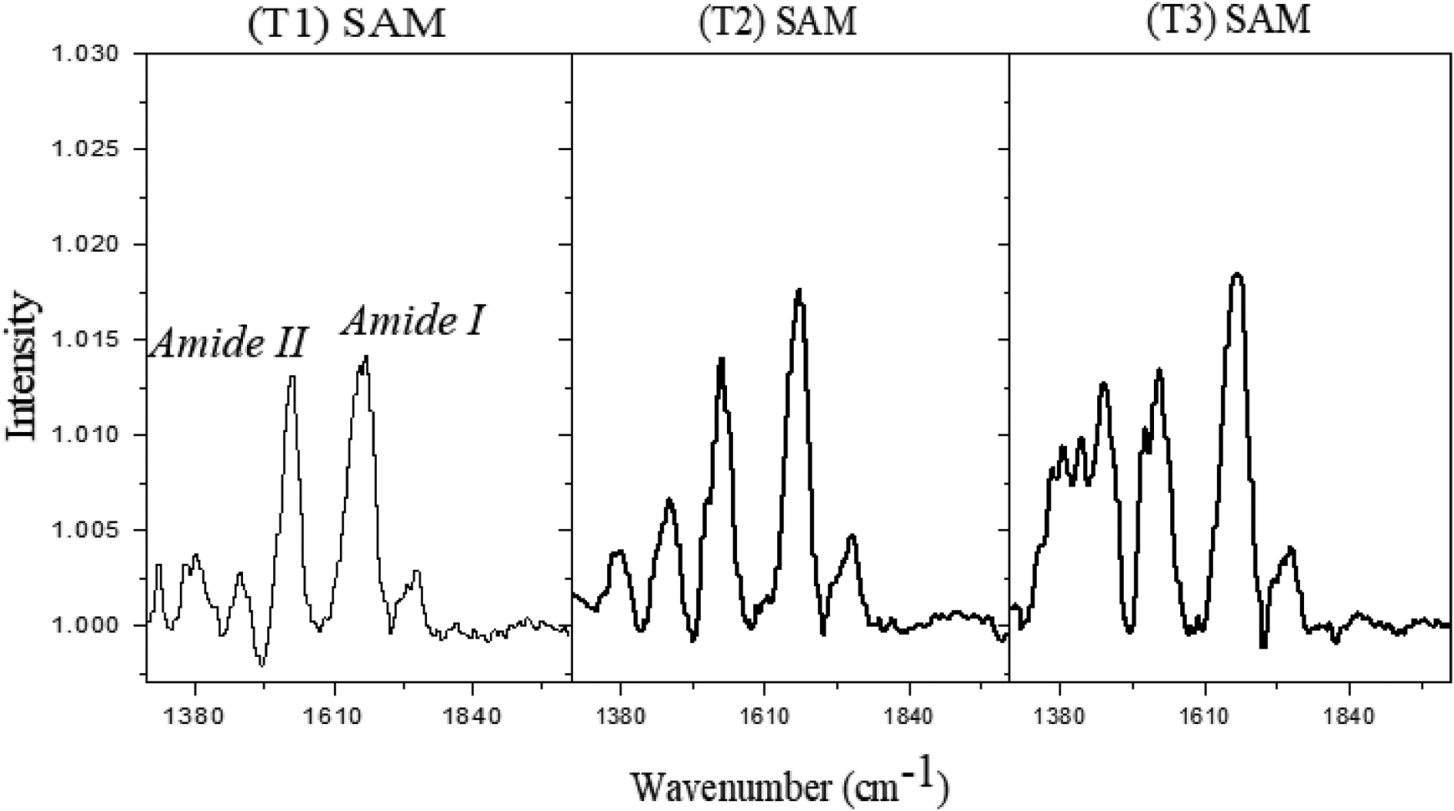
PM‐IRRAS spectra of the SAMs grown by **T1**, **T2**, and **T3**.

**TABLE 2:** Summary of the surface analysis data obtained from PM‐IRRAS.

Compound‐SAM	Amide I (cm^−1^)	Amide II (cm^−1^)	Tilt angle *γ* (deg, ±4)
**T1**	1661	1541	57
**T2**	1663	1541	55
**T3**	1660	1541	56

The main uncertainty arises from the scaling factor (K). As (K) could not be determined directly for the present compounds because of the limited amount of available material, a value of 1.67 was obtained from the average of three values previously determined for derivatives containing the same peptide backbone (K = 1.40, 1.75, and 1.85). Considering the full range of these values results in effective tilt‐angle ranges of approximately 53°–61°, 51°–58°, and 52°–59° for **T1**, **T2**, and **T3**, respectively. Thus, within the accuracy of the PM‐IRRAS analysis, no significant difference in average helix orientation can be established among the three SAMs.

The somewhat larger effective tilt angles previously reported for these assemblies compared with the BDP‐C_60_‐peptide assembly and hexapeptide **5** (43.7° and 48.5°, respectively) may nevertheless be consistent with a less constrained organization of the present monolayers. The additional Fc fragments grafted onto the BDP moiety could contribute to such an effect through increased steric requirements; however, this interpretation should be regarded as qualitative in view of the uncertainty of the absolute tilt‐angle determination.

#### 2.4.2 Ultrafast Electrochemistry

The SAMs grown by the target assemblies **T1**, **T2**, and **T3** on gold surfaces were evaluated by CV in order to confirm that the molecules were surface‐adsorbed on gold in the first place. Moreover, additional ultrafast electrochemical analyses were conducted, as previously described [33], in order to estimate the surface coverage and the electron transfer rate constants [40, 41, 42, 43]. The electrochemical analyses were performed using 0.3 M of TBAPF_6_ as electrolyte in DCM after 18 h of impregnation time for the grafting process of the target assemblies on gold ball microelectrodes. The obtained cyclic voltammograms displayed well‐resolved peaks with bell‐shaped behaviors and very low peak‐to‐peak separation (less than 10 mV), presenting useful data for the extraction of important parameters including the half‐wave potentials and the electron transfer rate constants [44]. The CVs of the target molecules **T1**, **T2**, and **T3** on the SAMs are presented in Figure 8 with their corresponding half‐wave potentials listed in Table 3.

**FIGURE 8:**
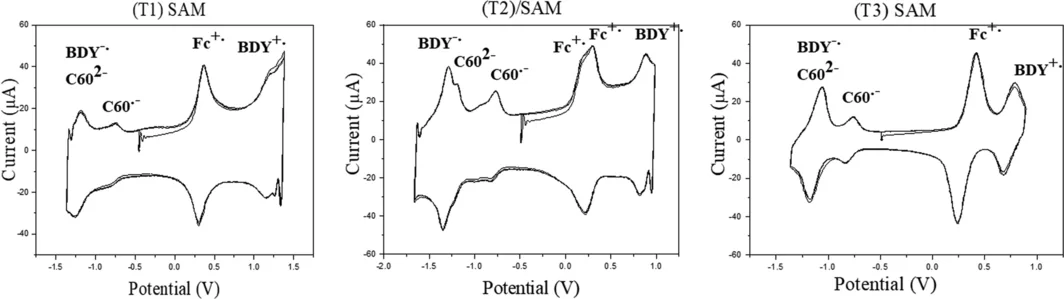
(Left) CV of **T1** adsorbed on a gold microelectrode at 5600 V·s^−1^; (middle) CV of **T2** adsorbed on a gold microelectrode at 5400 V·s^−1^; (right) CV of **T3** adsorbed on a gold microelectrode at 4600 V·s^−1^.

**TABLE 3:** Electrochemical half‐wave potentials (V vs. Fc^+^/Fc) of the target assemblies **T1**, **T2**, and **T3** obtained from CVs on grown SAMs on gold microelectrodes.

Compound	Potential (V vs. Fc^+^/Fc)				
	BDP^0/+^	Fc^0/+^	C_60_^0/−^	C_60_^−/2−^	BDP^0/−^
(**T1**) SAM	+1.05	+0.17	−0.88	−1.32	−1.32
(**T2**) SAM	+0.74	+0.15	−0.90	−1.30	−1.42
(**T3**) SAM	+0.62	+0.21	−0.90	−1.22	−1.22

The electrochemical signatures of the redox centers (BDP, C_60_, and Fc) within the target assemblies **T1**, **T2**, and **T3** on SAMs are in accordance with the obtained electrochemical data for the parent tetrads (**4a**‐**4c**) in solution, which confirms that our assemblies were successfully immobilized on the metallic surfaces. The standard potential separation values between the redox centers are very similar in solution and on gold surfaces. However, as clearly observed in Figure 8, the capacitive current of the **T1‐** and **T2**‐SAM is higher than the capacitive current of **T3**, suggesting that the SAM formed by the latter assembly might be more densely packed than the SAM obtained by the former assemblies.

The integration of the area under the cathodic or anodic peak that corresponds to the oxidation or reduction of the redox centers allows us to estimate the surface density of our assemblies on the gold microelectrodes using the same procedure as previously described. We found here coverages in the same range as previously found with structures having a similar backbone. These values are reported in Table S2 together with the capacitances per unit area, which are in line with those currently observed for SAMs. **T1** presents the lowest coverage in agreement with a larger steric hindrance of the ferrocene linkers (see estimation of the molecular footprint in Figure S49). The calculation of the electron transfer rate constants k_s_, corresponding to the target assemblies **T1**, **T2**, and **T3** on gold microelectrodes, was carried out in a similar manner to that described in our previous study [33]. Therefore, classical CVs of the different redox centers (Fc, BDP, and C_60_) by increasing the scan rates were recorded for each assembly. The recorded CVs of the Fc waves of each assembly as a function of the scan rates are displayed in Figure S48. Moreover, the plotted data of the peak current as the function of the scan rates for the three target molecules gave linear functions in all cases (Figure S48), which confirms the successful immobilization of our target assemblies on the gold microelectrodes. Finally, the extraction of k_s_ values was achieved by fitting ΔEp as the function of scan rates using reference curves produced by numerical simulations. The experimental and simulated data are presented in the same figure, and the extracted values of the electron transfer rates of each redox center are listed in Table 4.

**TABLE 4:** Calculated values of electron transfer rate constants k_S_ from each redox center for the SAMs formed by **T1**, **T2**, and **T3** assemblies on gold microelectrodes.

Compound‐SAM	Electron transfer rate constant k_s_ × 10^4^ (s^−1^)		
	BDP^0/+^	Fc^0/+^	C_60_^0/−^
**T1**	13	6	20
**T2**	9	6	13
**T3**	11	2	11

A general inspection of the electron transfer rate constants of the target assemblies **T1**‐**T3** shows that the obtained k_s_ values are higher than those previously reported for the C_60_ and BDP assemblies [33]. This difference may arise from a shorter equilibrium distance between the redox center and the electrode, or from a more folded (i.e., flexible) molecular organization, which could also be consistent with differences in surface organization or packing. These observations suggest that the flexible linker between the peptide and C_60_ may allow C_60_ to insert between the peptide chains, positioning it relatively close to the electrode. A similar situation may apply to BDP. These hypotheses are based on a through‐space electron transfer mechanism and do not consider any electronic contribution from the peptide backbone. In contrast, the k_s_ values for Fc are substantially higher for **T1** and **T2** than for **T3**, suggesting that the Fc moiety is located farther from the electrode in **T3**. A tentative representation of the corresponding molecular arrangements is proposed in Figure 9.

**FIGURE 9:**
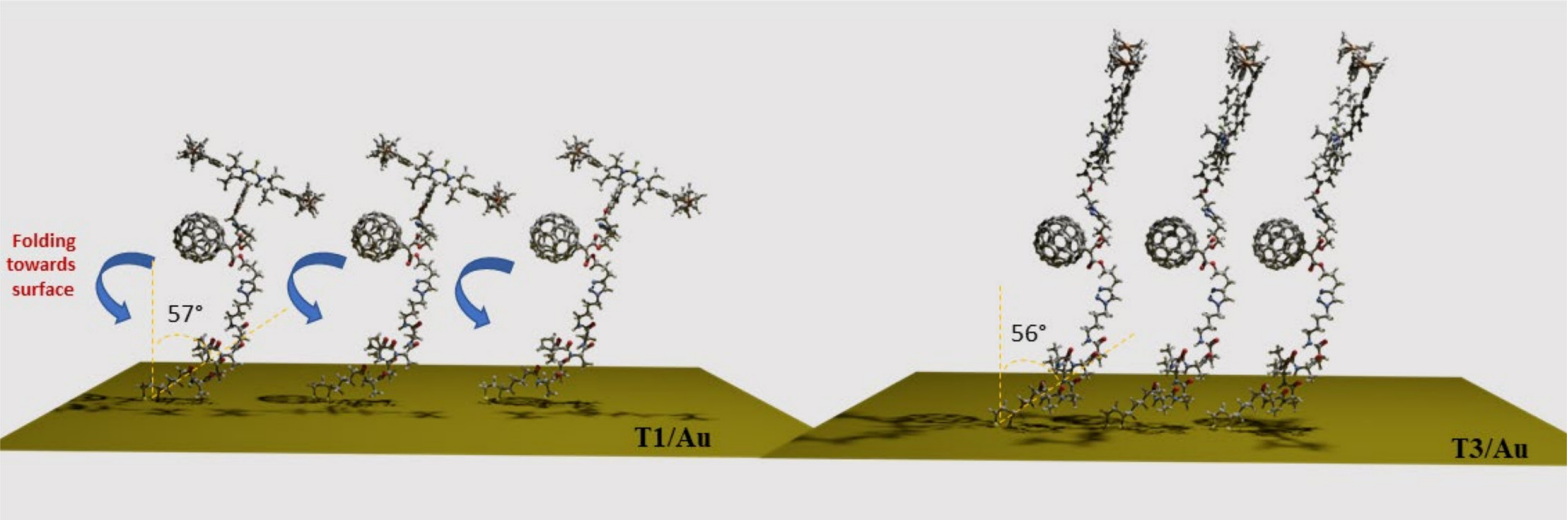
Schematic representation of the SAMs in the target assembly **T1**, showing the proposed folding of the alkyl chain between the peptide and the C_60_. The SAMs of target **T3** are denser and less prone to folding.

## 3 Conclusion

In summary, we have succeeded in the first place to efficiently synthesize and isolate complex molecular assemblies (Fc)_2_‐BDP‐C_60_ (**4a**‐**4c**) and (Fc)_2_‐BDP‐C_60_‐peptide (**T1‐T3**) in good yields using our previously reported synthetic strategy. The incorporation of Fc units onto the BODIPY core alters the photophysical properties of the resulting assemblies. The absorption properties are broader in the visible region, due to the extended π‐conjugation of the BODIPY core and to the presence of an ICT band from ferrocene to BODIPY, particularly in the case of **4a/T1** and **4b/T2**. In addition, all the (Fc)_2_‐BDP‐C_60_ assemblies exhibited a very strong quenching of fluorescence, which can be attributed to photo‐induced electron‐transfer processes involving the Fc and C_60_ units and the photoexcited chromophore BDP, as we have already demonstrated in a previous study. It should be noted that the peptide in these systems is electronically decoupled from the photoactive framework and therefore has no effect on the photophysical properties. These target assemblies (**T1**‐**T3**) were successively grafted on gold surfaces through the formation of SAMs, and then characterized by a set of electrochemical and surface analyses. The obtained results confirmed the successful immobilization of **T1**, **T2**, and **T3** assemblies on gold surfaces by the characteristic bands from PM‐IRRAS (amide I, amide II, C=O) and redox signatures (Fc, BDP, C_60_) from ultrafast electrochemical experiments. Moreover, the estimation of the tilt angle of the SAMs formed by these assemblies (**T1**, **T2**, and **T3**) using PM‐IRRAS revealed the formation of less vertically‐oriented SAMs compared with those formed by the C_60_‐peptide and BDP‐C_60_‐peptide previously reported. From ultrafast electrochemical experiments, we found here surface coverages in the same range as previously (see Table S2) in accordance with structures having a similar backbone, with calculated surface coverages estimated at 2.5 × 10^−11^, 3.4 × 10^−11^, and 5.2 × 10^−11^ mol·cm^−2^ for **T1**, **T2**, and **T3**, respectively. **T1**, where the Fc unit is in the beta position on the BDP core, presents the lowest coverage, in agreement with an increase in steric hindrance caused by this arrangement. From these findings, it is clear that the presence of the Fc units on the BDP core may influence intermolecular packing interactions within the monolayers, modifying the molecular organization of the resulting SAMs, with alterations in surface coverage and orientation.

The estimation of the heterogeneous electron transfer rate constants of **T1**, **T2**, and **T3** SAMs from ultra‐fast electrochemical experiments revealed higher k_s_ values corresponding to the electron transfer from the BDP or C_60_ entities to the metallic surface, in comparison with the BDP‐C_60_‐peptide previously reported. This could be attributed to folding of the (Fc)_2_‐BDP‐C_60_ part towards the surface, allowing (Fc)_2_‐BDP‐C_60_ to be inserted between the peptide chains and therefore positioned rather close to the surface.

Finally, these systems appear to be promising candidates for the development of photoelectrochemical (PEC) cells for photocurrent generation, as already demonstrated for related systems. Indeed, these molecular assemblies exhibit several attractive features, including broad absorption covering both the visible and near‐infrared regions, together with multistep photoinduced electron transfer processes, both features being associated with the grafting of ferrocene units on the BODIPY core. These characteristics are expected to favor light harvesting and charge separation, and could therefore lead to improved photocurrent generation.

## Funding

This work was supported by the LabEx CHARMMMAT ANR‐11‐LABS‐0039, the CNRS, the University of Versailles St‐Quentin‐en‐Yvelines, the University of Paris‐Saclay, and the MESRI.

## Conflicts of Interest

The authors declare no conflicts of interest.

## Supporting information


**Scheme S1.** Synthesis of Fc‐BDP derivatives **1b**‐**2b**.
**Figure S1:** ^1^H NMR of compound **1b**.
**Figure S2:** ^13^C NMR of compound **1b**.
**Figure S3:** ^19^F NMR of compound **1b**.
**Figure S4:** ^11^B NMR of compound **1b**.
**Figure S5:** ^1^H NMR of compound **2b**.
**Figure S6:** ^13^C NMR of compound **2b**.
**Figure S7:** ^19^F NMR of compound **2b**.
**Figure S8:** ^11^B NMR of compound **2b**.
**Figure S9:** ^1^H NMR of compound **2c**.
**Figure S10:** ^13^C NMR of compound **2c**.
**Figure S11:** ^19^F NMR of compound **2c**.
**Figure S12:** ^11^B NMR of compound **2c**.
**Figure S13:** ^1^H NMR of compound **4b**.
**Figure S14:** psc70142‐sup‐0001‐Supplementary_Material.docx. ^13^C NMR of compound **4b**.
**Figure S15:** ^19^F NMR of compound **4b**.
**Figure S16:** psc70142‐sup‐0001‐Supplementary_Material.docx. ^11^B NMR of compound **4b**.
**Figure S17:** ^1^H NMR of compound **4c**.
**Figure S18:** ^13^C NMR of compound **4c**.
**Figure S19:** ^19^F NMR of compound **4c**.
**Figure S20:** ^11^B NMR of compound **4c**.
**Figure S21:** ^1^H NMR spectra in C_5_D_5_N (300 MHz, 298 K) of the target assembly **T1**. The peaks labelled with an asterisk (* and #) are due to ligand residues and water, respectively.
**Figure S22:** ^1^H NMR spectra of **T1** (enlargement of the 6.5–10 ppm region). The peaks labelled with an asterisk * are due to ligand residues.
**Figure S23:** ^1^H NMR spectra of **T1** (enlargement of the 6.5–10 ppm region). The peaks labelled with an asterisk (* and #) are due to ligand residues and water, respectively.
**Figure S24:** ^11^B NMR of compound **T1**.
**Figure S25:** Mass spectrum of compound **T1**.
**Figure S26:** Theoretical and experimental [M + H]^+^ masses of compound **T1**.
**Figure S27:** ^1^H NMR spectra in C_5_D_5_N (300 MHz, 298 K) of the target assembly **T2**.
**Figure S28:** ^1^H NMR spectra of **T2** (enlargement of the 6.5–10 ppm region).
**Figure S29:** ^1^H NMR spectra of **T2** (enlargement of the 0–5.5 ppm region).
**Figure S30:** ^19^F NMR spectra of **T2**.
**Figure S31:** ^11^B NMR spectra of **T2**.
**Figure S32:** Mass spectrum of compound **T2**.
**Figure S33:** Mass difference report on [M^+•^ + H]^2+^ masses of compound **T2**.
**Figure S34:** ^1^H NMR spectra in C_5_D_5_N (300 MHz, 298 K) of the target assembly **T3**.
**Figure S35:** ^1^H NMR spectra of **T3** (enlargement of the 6.5–10 ppm region).
**Figure S36:** ^1^H NMR spectra of **T3** (enlargement of the 0.6–5.2 ppm region).
**Figure S37:** ^19^F NMR spectra of **T3**.
**Figure S38:** ^11^B NMR spectra of **T3**.
**Figure S39:** Mass spectrum of compound **T3**.
**Figure S40:** Theoretical and experimental [M + Na]^+^ masses of compound **T3**.
**Figure S41:** Normalized absorption spectra of (Fc)_2_‐BDP **1a** and BODIPY model compound **1d**.
**Figure S42:** Normalized absorption spectra of (Fc)_2_‐BDP **1b** and DSBDP.
**Figure S43:** Normalized absorption spectra of (Fc)_2_‐BDP **1c** and BODIPY model compound **1e**.
**Figure S44:** Normalized absorption spectra of (Fc)_2_‐BDP **1a**‐**1c** and fullerene platform **3**.
**Figure S45:** Normalized absorption spectra of (Fc)_2_‐BDP‐C_60_ conjugates **4a**‐**4c** and the molecular assemblies (Fc)_2_‐BDP‐C_60_‐peptide **T1**‐**T3**. Inset: Absorption spectrum of hexapeptide **5**.
**Figure S46:** Emission spectra of compounds **1b**‐**1c** and **4b**‐**4c**, recorded in DCM. Top: Reference molecules **BDP** (λexc = 495 nm) and **DSBDP** (λexc = 605 nm) and (Fc)_2_‐BDP precursors **1b** (λexc = 550 nm) and **1c** (λexc = 590 nm). Bottom: **DSBDP** (λexc = 605 nm) and (Fc)_2_‐BDP‐C_60_ target assemblies **4b** (λexc = 550 nm) and **4c** (λexc = 605 nm).
**Table S1:** Absorption and fluorescence data of the triads **1a‐1c** and tetrads **4a‐4c**, recorded in DCM.
**Figure S47:** Cyclic voltammograms of the triad **1a‐1c** in anhydrous and oxygen‐free DCM, 0.1 M of *n*‐Bu_4_NPF_6_, pseudo‐reference electrode: Ag wire, working electrode: Pt, auxiliary electrode: Pt, scan rate: 100 mV.s^−1^.
**Figure S48: (left)** Scan‐rate dependence of the cyclic voltammograms; **(middle)** peak current as a function of scan rate; **(right)** Experimental (dots) and simulated (lines) peak to peak potential variation versus scan rates of the target assemblies SAMs on gold microelectrodes. Deviations from simulations at larger scan rates results from instrumental distortions by the potentiostat (bandwidth limitation).
**Table S2:** Molecular coverages Γ and capacitances per surface areas C.
**Figure S49:** Estimated molecular footprint of (Fc)_2_‐BDP‐C_60_‐peptides assemblies **T1**, **T2** and **T3** assuming that these structures are in extended conformations and that the hexapeptide adopts an helical conformation.

## Data Availability

The data that support the findings of this study are available from the corresponding author upon reasonable request.
