# Supplementary material for: Self‐Assembled Monolayers on Gold Surfaces of Ferrocene‐BODIPY‐Fullerene‐Helical Peptide Conjugates: Studies of Electron Transfer Processes by Ultra‐Fast Electrochemistry
Source: J Pept Sci. 2026 Oct 27;32(12):e70142. doi: 10.1002/psc.70142 (PMC13714168; doi:10.1002/psc.70142)
Supplement: Supplementary file 1 [file PSC-32-e70142-s001.docx]

Self-Assembled Monolayers on Gold Surfaces of Ferrocene-BODIPY-Fullerene-Helical Peptide Conjugates: Studies of Electron Transfer processes by Ultra-fast Electrochemistry.

# Jad Rabah,^a^ Anne Vallée,^a,c^ Hélène Fensterbank,^a^ Minh-Huong Ha-Thi,^b^ Rachel Méallet,^b^ Souhir Boujday,^c^ Emmanuel Maisonhaute,^d*^ Karen Wright,^a*^ and Emmanuel Allard^a*^

# ^a^ Université Paris-Saclay, UVSQ, CNRS, Institut Lavoisier de Versailles, 78 000, Versailles, France, e-mail: [emmanuel.allard@uvsq.fr](mailto:emmanuel.allard@uvsq.fr) and [karen.wright@uvsq.fr](mailto:karen.wright@uvsq.fr)

# ^b^ Université Paris-Saclay, CNRS, Institut des Sciences Moléculaires d’Orsay, 91 405, Orsay, France.

# ^c^ Laboratoire de Réactivité de Surface, Sorbonne Université, France.

# ^d^ Laboratoire Interfaces et Systèmes Electrochimiques, Sorbonne Université, CNRS, 4 place Jussieu, 75005 Paris, France

e-mail : [emmanuel.maisonhaute@upmc.fr](mailto:emmanuel.maisonhaute@upmc.fr)

- Materials and Methods	**pages S3-S5**
- Synthesis and NMR spectra of compound **1b**	**pages S6-S8**
- Synthesis and NMR spectra of compound **2b	pages S9-S11**
- Synthesis and NMR spectra of compound **2c	pages S12-S14**
- Synthesis and NMR spectra of compound **4b	pages S15-S17**
- Synthesis and NMR spectra of compound **4c	pages S18-S20**
- Synthesis, NMR and Mass spectra of compound **T1	pages S21-S24**
- Synthesis, NMR and Mass spectra of compound **T2	pages S25-S29**
- Synthesis, NMR and Mass spectra of compound **T3	pages S30-S34**
- Absorption spectra of compounds **1a**-**1c**, **3**	**pages S35-S36**

and models compounds **1d**, **1e** and **DSBDP**

- Absorption spectra of **(**Fc)_2_-BDP-C_60_	**page S37**

and **(**Fc)_2_-BDP-C_60_-peptide assemblies

- Fluorescence emission spectra of compounds **1b**-**1c** and **4b**-**4c**	**page S38**
- Absorption and Fluorescence data of compounds **1a**-**1c** and **4**a-**4**c	**page S39**
- CVs of compounds **1a-1c	page S39**
- CVs of compounds **T1-T3** SAMs on gold microelectrode	**page S40**
- Molecular coverages Γ and capacitances per surface areas C	**page S40**
- Estimated molecular footprint of **T1**-**T3** assemblies on gold	**page S41**

**Experimental Section**

**Materials and methods**

***General methods*.** All reagents were used directly without further purification. Fullerene C_60_ (purity 99.5+%) was purchased from MTR Ltd. Dichloromethane (DCM) was dried and distilled over CaH_2_ prior to use. All other solvents were used as received. Column chromatography: silica gel 60 (0.040-0.063 mm) was purchased from E. Merck. Thin layer chromatography (TLC) was performed on aluminum sheets coated with silica gel 60 F_254_ from E. Merck and visualized by UV light. ^1^H, ^13^C, ^19^F and ^1^B NMR spectra were recorded using a Brucker spectrometer at respectively 300MHz, 75.5 MHz, 282 MHz and 96 MHz in C_5_D_5_N or CDCl_3_. Chemical shifts (δ) were referenced to the internal solvent (^1^H and ^13^C NMR). High Resolution ESI-MS mass spectra were obtained with a Waters Xevo Qtof.

The compounds **1a**, **1c**, **1d**, **1e**, **DSBDP**, **2a**, methanofullerene C_60_ platform **3** and hexapeptide **5** were prepared according to our previously reported procedures.^[^^[1]]^ The ligand N,N’,N’’-tris(2-dibenzylaminoethyl)amine (hexabenzyltren) was synthesized in two steps from tris(2-aminoethyl)amine as previously described.^[1a,^ ^[2]]^

***Steady-State Absorption and Fluorescence Spectroscopy.*** Optical measurements were performed in 10 mm path length quartz cuvettes. Absorption spectra were recorded using a Shimadzu UV-visible spectrophotometer UV-2600 in transmission mode. The emission spectra were recorded on diluted solutions (to avoid reabsorption artefacts), using a Fluoromax-4 spectrofluorimeter (Jobin-Yvon; Horiba). The signals were corrected from lamp fluctuations and apparatus response functions.

***Preparation of SAMs.*** For the immobilization of our target and reference assemblies on surfaces, we used 11 mm × 11 mm ArrandeeTM (Werther, Germany) gold plates, which consist of a borosilicate glass substrate (0.7 +/- 0.1 mm), a thin chromium interlayer (2.5 +/- 1.5 nm) and a gold layer (250 +/- 50 nm). The gold coated substrates were cleaned by annealing in a butane flame to ensure good crystallinity of the topmost layers and rinsed in a bath of absolute ethanol for 15 min before adsorption. All the target compounds were individually immobilized on gold surfaces by exposing a gold plate to a 10^-4^ M oxygen-free solution of the compounds in dichloromethane, in the dark for 18 hours at room temperature under argon atmosphere. The modified surfaces were then rinsed with DCM and water and finally dried with a stream of nitrogen.

The SAMs on the gold ball microelectrodes for ultrafast voltammetry (see below) were prepared by incubation for 18 hours on the clean electrode in a 0.1 mM solution of the molecule in DCM. The electrodes were rinsed and left for 30 minutes in neat DCM prior performing electrochemical measurements.

***PM-IRRAS.*** The analyses were performed in air with the crystal placed in the external beam of a Fourier transform infrared Nicolet 5700 spectrometer. All reported spectra were recorded at 8 cm^-1^ resolution by co-addition of 128 scans; using a modulation of polarization enabled us to perform rapid analyses of the samples after immersion without purging the atmosphere or requiring a reference spectrum.^[^^[3]]^

The molecular orientation of the helical peptide was determined from the Amide I/Amide II ratio in the RAIRS spectrum under the assumption of uniform orientation of the helix axis around the surface normal^[^^[4]]^ according to the following formula:^[^^[5]]^

$$\frac{I_{1}}{I_{2}}=K \frac{\left( 3{cos}^{2}\gamma-1 \right)\left( 3{cos}^{2}\theta_{1}-1 \right)+2}{\left( 3{cos}^{2}\gamma-1 \right)\left( 3{cos}^{2}\theta_{2}-1 \right)+2}$$

Where Ii, γ, and θi (i = 1 or 2 corresponding to amide I or amide II) represent the observed absorbance, the tilt angle of the helical axis from the surface normal, and the angle between the transition moment and the helix axis, respectively. The value of θ_1_ was set to be 39° while θ_2_ was taken between 75° for an α-helix and 83° for a 3_10_-helix, according to the literature.^[^^[6]]^

K is the scaling factor related to the intrinsic “oscillator strength” of the amide I and amide II vibrational mode, which account for the amide I/II absorbance ratios experimentally determined for the respective helical peptides in the solid phase.

***Ultrafast electrochemistry.*** The CVs were performed in a standard three-compartment electrochemical cell under argon atmosphere with an ultrafast Potentiostat (allowing on-line ohmic drop compensation) using two platinum coils as reference and counter electrodes, and the modified gold ball microelectrodes as working electrode in the presence of 0.3 M of TBAPF_6_ as electrolyte in DCM. Gold ball ultra-microelectrodes were produced as described previously.^[^^[7]]^ Briefly, about 5 cm of a 25 µm diameter gold wire (goodfellow) is soldered onto a larger copper wire. The wire is inserted in a glass pipet so that ca 2-3 mm of gold protrudes from the wire. The glass is then melted by inserting the assembly in a heating coil. Afterwards, the protruding wire is melted carefully in a butane flame so as to obtain a small ball. These electrodes present the advantage to produce reproducible results when adsorbing molecular systems. They can be regenerated by passing the electrode in the flame to remove any adsorbant.

- Estimation of the SAMs’ surface coverage (Γ):

Integration of the area under the cathodic or anodic peak, owing to the reductions or oxidations of the redox counterparts, allows us to calculate the surface coverage of our assemblies on the gold microelectrodes. The surface coverage Γ (mol cm^−2^) of modified gold microelectrodes is estimated using the following equation^[^^[8]]^:

Γ = Q /nFA

Where Q (C) is the charge for oxidation of redox center calculated by integration of the area under the oxidation or reduction peak of the corresponding redox center, F is the Faraday constant, and A (cm^2^) is the electrode area. The active electrode area is calculated using the Randles−Sevcik equation^[8]^:

i_p_ = 268600n^3/2^AD^1/2^Cν^1/2^

Where i_p_ is the peak current, n is the number of electrons exchanged (n = 1), A (cm^2^) is the area, D (cm^2^ s^−1^) and C (mol cm^−3^) are the diffusion coefficient and concentration of the redox couple used as a probe, and v (V s^−1^) is the scan rate. Ferrocene (1 mM) in DCM + 0.1 M TBAPF_6_ was used as the redox probe. Its relative diffusion coefficient in this medium is known to be D = 2.2 ×10^−5^ cm^2^ s^−1^.^[^^[9]]^

- Estimation of the SAMs’ electron transfer rate constants (k_s_):

The heterogeneous rate constant between the redox centers and the electrode was determined through the investigation of signatures of the redox counterparts by increasing the scan rate. The k_S_ values were then extracted through fitting the peak to peak potential variation (ΔEp) with the scan rate upon the Laviron theory using reference curves produced by numerical simulations.^[^^[10]]^

**Synthetic procedures**

**Scheme S1.** Synthesis of Fc-BDP derivatives **1b**-**2b**.

*Synthesis of compound* ***1b***

To a 25 mL flask under argon was added starting material **BDP** (100 mg, 0.23 mmol), **formylferrocene** (291 mg, 1.36 mmol), and piperidine (0.35 mL, 2.27 mmol) in dry acetonitrile (7 mL). Argon was bubbled through the solution for 30 min. The mixture was refluxed overnight at 100^o^C under argon. The mixture was cooled to room temperature and concentrated under reduced pressure. After purification by column chromatography on silica gel (chloroform/Et_2_O 96:4), a blue solid was obtained (yield = 27%). **^1^H NMR (300 MHz, CDCl_3_)** δ: 1.15 (t, *J* = 7.4 Hz, 6H; H_B_), 1.36 (s, 6H; H_D_), 2.16 (bs, 1H; OH), 2.58 (q, *J* = 7.3 Hz, 4H; H_C_), 4.03 (bs, 2H; H_H_), 4.13-4.18 (m, 2H; H_G_), 4.23 (s, 10H; H_Fc_), 4.40-4.43 (m, 4H; H_Fc_), 4.63-4.66 (m, 4H; H_Fc_), 7.03 (d, *J* = 8.6 Hz, 2H; H_E_ or H_F_), 7.12 (d, *J* = 16.5 Hz, 2H; H_A_), 7.20 (d, *J* = 8.6 Hz, 2H; H_E_ or H_F_), 7.38 (d, *J* = 16.5 Hz, 2H; H_A_); **^19^F NMR (282 MHz, CDCl_3_**) δ: -138.70 (q, *J = 34.4 Hz*); **^11^B NMR (96 MHz, CD_3_CN)** δ: 90.64 (t, *J= 34.5 Hz*); **^13^C NMR (75 MHz, CDCl_3_)** δ 11.8 (-CH_3_), 14.4 (CH_2_-CH_3_), 18.4 (-CH_2_-CH_3_), 61.5 (-CH_2_-OH), 68.0 (C_Fc_), 69.3 (-CH_2_-O-), 69.8 (C_Fc_), 70.3 (C_Fc_), 83.4 (C_A_), 115.1 (C_BDP-arom_), 117.8 (C_styryl_), 129.0, 130.0, 133.3, 133.3 (C_BDP-arom_), 135.9 (C_A_), 137.0, 138.3, 150.0, 159.1 (C_BDP-arom_); **HRMS (ESI+)** m/z calcd for C_47_H_47_BF_2_Fe_2_N_2_O_2_: 832.2386 [M +^.^], found: 832.2405.

**Figure S1.** ^1^H NMR of compound **1b** **Figure S2.** ^13^C NMR of compound **1b** **Figure S3.** ^19^F NMR of compound **1b**

**Figure S4.** ^11^B NMR of compound **1b**

*Synthesis of compound* ***2b***

To a 10 mL flask under argon was added PPh_3_ (48 mg, 0.185 mmol), NBS (32 mg, 0.185 mmol), and **compound 1b** (60 mg, 0.072 mmol) in dry DCE (5 mL) at 0^o^C. The mixture was stirred at 0^o^C under argon till the complete consumption of the starting material. Then 2 mL of NaN_3_ solution (25% w/v aq) and 2 drops of Aliquat 336 were added, and the mixture was stirred overnight at 80 ^o^C. Then, the solution was washed with 20 mL of water, extracted with DCM, dried with MgSO_4_, filtered on cotton, and concentrated under reduced pressure. After purification by column chromatography on silica gel (toluene), a blue solid was obtained (yield = 67%). **^1^H NMR (300 MHz, CDCl3)** δ: 1.15 (t, *J* = 7.4 Hz, 6H; H_B_), 1.36 (s, 6H; H_D_), 2.57 (q, *J* = 7.3 Hz, 4H; H_C_), 3.68 (t, *J = 4.7 Hz*, 2H; H_H_), 4.18-4.27 (m, 12H; H_Fc_, H_G_), 4.35-4.46 (m, 4H; H_Fc_), 4.60-4.68 (m, 4H; H_Fc_), 7.04 (d, *J* = 8.6 Hz, 2H; H_E_ or H_F_), 7.11 (d, *J* = 16.5 Hz, 2H; H_A_), 7.21-7.22 (m, 2H_,_ H_E_ or H_F_), 7.37 (d, *J* = 16.5 Hz, 2H; H_A_); **^19^F NMR** (282 MHz, CDCl_3_) δ: -138.78 (q, *J* = 32.1 Hz); **^11^B NMR** (96 MHz, CDCl_3_) δ: 1.29 (t, *J* = 34.2 Hz); **^13^C NMR (75 MHz, CDCl_3_)** δ 11.9 (-CH_3_), 14.4 (-CH_2_-CH_3_), 18.4 (-CH_2_-CH_3_), 50.4 (-CH_2_-CH_2_-N_3_), 67.1, 68.03 (C_Fc_), 69.9 (CH_2_-CH_2_-O-), 70.2 (C_Fc_), 83.4 (C_vinyl_), 115.5 (C_arom-BDP_), 117.8 (C_vinyl_), 129.4, 130.1, 133.2, 133.4, 133.5 (C_arom-BDP_), 135.9 (C_vinyl_), 137.0, 138.3, 150.1, 158.7 (C_arom-BDP_); **HRMS (ESI+)** m/z calcd for C_47_H_46_BF_2_Fe_2_N_5_O: 857.2451 [M^+.^], Found: 857.2466.

 **Figure S5.** ^1^H NMR of compound **2b**

**Figure S6.** ^13^C NMR of compound **2b**

**Figure S7.** ^19^F NMR of compound **2b**

 **Figure S8.** ^11^B NMR of compound **2b**

*Synthesis of compound* ***2c***

To a 10 mL flask under argon was added PPh_3_ (96 mg, 0.36 mmol), NBS (60 mg, 0.34 mmol), and **compound 1c** (45 mg, 0.044 mmol) in dry DCE (3 mL) at 0^o^C. The mixture was stirred at 0^o^C under argon till the complete consumption of the starting material. Then 0.7 mL of NaN_3_ solution (25% w/v aq) and 2 drops of Aliquat 336 were added, and the mixture was stirred overnight at 80 ^o^C. Then, the solution was washed with 20 mL of water, extracted with DCM, dried with MgSO_4_, filtered on cotton, and concentrated under reduced pressure. After purification by column chromatography on silica gel (DCM/Pentane 7/3), a dark green solid was obtained (yield = 77%). **^1^H NMR (300 MHz, CDCl3)** δ: 1.18 (t, *J* = 7.4 Hz, 6H; H_B_), 1.39 (s, 6H; H_D_), 2.53-2.69 (m, 4H; H_C_), 3.69 (t, *J = 4.7 Hz,* 2H; H_H_), 4.22-4.33 (m, 16H; H_G,_ H_Fc_), 4.48-4.60 (m, 4H; H_Fc_), 7.06 (d, *J* = 8.5 Hz, 2H; H_E_ or H_F_), 7.21-7.24 (m, 4H; H_A_, H_E_ or H_F_), 7.51 (d, *J* = 8.2 Hz, 4H; H_arom_), 7.59 (d, *J* = 8.3 Hz, 4H; H_arom_), 7.82 (d, *J* = 16.7 Hz, 2H; H_A_); **^19^F NMR (282 MHz, CDCl_3_**) δ: -138.63 (q, *J = 34.4 Hz*); **^11^B NMR (96 MHz, CDCl_3_)** δ 1.25 (t, *J= 34.5 Hz*); **^13^C NMR (75 MHz, CDCl_3_)** δ 11.9 (-CH_3_), 14.2 (-CH_2_-CH_3_), 18.5 (-CH_2_-CH_3_), 50.4 (-CH_2_-N_3_), 67.1 (C_Fc_), 69.0 (-CH_2_-O), 70.1 (C_Fc_), 71.6 (C_Fc_), 86.2 (C_vinyl_), 90.4 (C_alkyne_), 115.3 (C_BDP-arom_), 120.6 (C_vinyl_), 124.2, 127.3, 128.8, 130.0, 131.8, 133.8, 133.2 (C_BDP-arom_), 135.2 (C_vinyl_), 136.7, 138.8, 139.3, 150.2, 158.9 (C_arom-BDP_); **HRMS (ESI+)** m/z calcd for C_63_H_54_BF_2_Fe_2_N_5_O: 1057.3077 [M^+.^], Found: 1057.3113.

 **Figure S9.** ^1^H NMR of compound **2c** **Figure S10.** ^13^C NMR of compound **2c** **Figure S11.** ^19^F NMR of compound **2c** **Figure S12.** ^11^B NMR of compound **2c**

*Synthesis of compound* ***4b***

To a 25 mL flask under argon was added methanofullerene platform **3** (67 mg, 0.065 mmol) in dry DCM (10 mL). Argon was bubbled through the solution for 30 minutes, then compound **2b** (40 mg, 0.045 mmol), DIPEA (8 μL, 0.045 mmol), and CuBr (7 mg, 0.045 mmol) were added. The mixture was stirred overnight at room temperature under argon. The solution was washed with 40 ml EDTA (0.05 M), then 20 mL of water, extracted with chloroform, dried with MgSO_4_, filtered on cotton, and concentrated under reduced pressure. After purification by column chromatography on silica gel (chloroform/Et_2_O 95/5), a blue solid was obtained (yield = 85%). **NMR ^1^H** (300 MHz, CDCl_3_) δ: 0.16 (s, 9H; H_n_), 1.13 (t*, J = 7.5 Hz*, 6H; H_B_), 1.31 (s, 6H; H_D_), 2.07 (qt, *J= 6.5 Hz*, 2H; H_j_), 2.32 (qt, *J= 6.9 Hz*, 2H; H_d_), 2.44 (t, *J = 7.0 Hz*, 2H; H_k_), 2.55 (q, *J= 7.5 Hz*, 4H, H_C_), 2.97 (t, *J= 7.3 Hz*, 2H; Hc), 4.17-4.28 (s, 10H; H_Fc_), 4.37-4.48 (m, 6H; H_Fc_ and H_H_), 4.54-4.66 (m, 8H; H_Fc_, He, Hi), 4.81 (t, *J = 4.8 Hz*, 2H; H_G_), 6.99 (d, *J = 8.5 Hz*, 2H; H_arom_), 7.09 (d, *J = 16.4 Hz*, 2H; H_A_), 7.20 (d, *J = 8.5 Hz*, 2H; H_arom_), 7.35 (, *J = 16.4 Hz*, 2H; H_A_), 7.62 (s, 1H; H_a_); **^13^C NMR (75 MHz, CDCl_3_)** δ: 0.3 (C_n_), 11.9 (C_D_), 14.4 (C_B_), 16.7 (C_k_), 18.5 (C_C_), 22.1 (C_c_), 27.8, 28.2 (C_d_, C_j_), 49.7 (C_H_), 52.2 (C_g_), 66.0 (C_e_, C_i_), 66.5 (C_G_), 68.0, 69.9, 70.1, 71.6 (C_Fc_, C_sp3-C60_), 83.3, 86.2 (C_m_), 105.1 (C_l_), 115.1 (C_arom-BDP_), 117.7 (C_vinyl_), 122.6 (C_a_), 128.3, 129.2, 129.9, 130.2, 133.2 (C_arom-BDP_), 136.0 (C_vinyl_), 136.5, 138.1, 139.1, 139.2 (C_arom-BDP_, C_60_, C_sp2_), 141.12, 141.15, 141.9, 142.1, 142.3, 142.33, 143.1, 143.13, 143.2, 144.0, 144.7, 144.78, 144.79, 144.82, 145.05, 145.1, 145.2, 145.26, 145.3, 145.4, 145.42, 146.6 (C_60_, C_sp2_), 150.1, 158.3, 163.7 (C_arom-BDP_), 163.8 (C_f_, C_h_); **^19^F NMR** (282 MHz, CDCl_3_) δ: -138.70 (q); **^11^B NMR** (96 MHz, CDCl_3_) δ: 1.24 (t, *J= 34.5 Hz*); **HRMS ESI (+)** calcd for C_123_H_68_BF_2_Fe_2_N_5_O_5_Si: 1884.3795 [M+^.^], Found: 1884.3767.

 **Figure S13.** ^1^H NMR of compound **4b**

 **Figure S14.** ^13^C NMR of compound **4b** **Figure S15.** ^19^F NMR of compound **4b**

 **Figure S16.** ^11^B NMR of compound **4b**

*Synthesis of compound* ***4c***

To a 25 mL flask under argon was added methanofullerene platform **3** (60 mg, 0.058 mmol) in dry DCM (10 mL). Argon was bubbled through the solution for 30 minutes, then compound **2c** (45 mg, 0.042 mmol), DIPEA (7 μL, 0.042 mmol), and CuBr (6 mg, 0.042 mmol) were added. The mixture was stirred overnight at room temperature under argon. The solution was washed with 40 ml EDTA (0.05 M), then 20 mL of water, extracted with DCM, dried with MgSO_4_, filtered on cotton, and concentrated under reduced pressure. After purification by column chromatography on silica gel (chloroform/Et_2_O 95/5), a dark green solid was obtained (yield = 80%). **NMR ^1^H** (300 MHz, CDCl_3_) δ: 0.16 (s, 9H; H_n_), 1.16 (t, *J = 7.3 Hz*, 6H; H_B_), 1.35 (s, 6H; H_D_), 2.07 (qt, *J = 6.7 Hz*, 2H; H_j_), 2.26-2.37 (m, 2H; H_d_), 2.45 (t, *J* = 6.9 Hz, 2H; H_k_), 2.52-2.68 (m, 4H; H_C_), 2.97 (t, *J= 7.1 Hz*, 2H; H_c_), 4.18-4.34 (m, 14H, H_Fc_), 4.45 (t, *J= 5 Hz*, 2H, H_H_), 4.41-4.68 (m, 8H; H_Fc_, H_e_, H_i_), 4.81 (t, *J= 5 Hz*, 2H; H_G_), 7.02 (d, *J = 8.2 Hz*, 2H; H_arom_), 7.14-7.21 (m, 4H; H_arom,_ H_A_), 7.51 (d, *J = 8.2 Hz*, 4H; H_arom_), 7.58 (d, *J = 8.2 Hz*, 4H; H_arom_), 7.62 (s, 1H; H_a_), 7.82 (d, *J = 16.8 Hz*, 2H; H_A_); **^13^C NMR (75 MHz, CDCl_3_)** δ: 0.3 (C_n_), 11.9 (C_D_), 14.2 (C_B_), 16.7 (C_k_), 18.5 (C_C_), 22.1 (C_c_), 27.8, 28.2 (C_d_, C_j_), 49.7 (C_H_), 52.1 (C_g_), 65.3, 66.0 (C_e_, C_i_), 66.5 (C_G_), 69.0, 70.1, 71.6 (C_Fc_, C_sp3-C60_), 86.2 (C_m_), 90.4 (C_alkyne_), 105.1 (C_l_), 115.3 (C_E_ or C_F_), 120.5 (C_vinyl_), 122.6 (C_a_), 124.2, 127.3, 128.3 129.9 (C_arom-BDP_), 130.1, 131.8, 133.8, 134.2 (C_arom-BDP_), 135.3 (C_vinyl_), 136.7, 138.3 (C_arom-BDP_), 139.1, 139.2 (C_60_, C_sp2_), 141.12, 141.15, 141.9, 142.1, 142.3, 142.33, 143.1, 143.13, 143.2, 144.0, 144.7, 144.8, 144.87, 145.2, 145.3, 145.4, 146.6 (C_60_, C_sp2_), 150.3, 158.5, 161.0, 163.7 (C_arom-BDP_), 163.8 (C_f_, C_h_); **^19^F NMR** (282 MHz, CDCl_3_) δ: -138.59 (q); **^11^B NMR** (96 MHz, CDCl_3_) δ: 1.23 (t, *J = 34.5 Hz*). **HRMS ESI (+)** calcd for C_139_H_76_BF_2_Fe_2_N_5_O_5_Si: 2084.4424 [M+^.^], Found: 2084.4473.

 **Figure S17.** ^1^H NMR of compound **4c**

 **Figure S18.** ^13^C NMR of compound **4c**

 **Figure S19.** ^19^F NMR of compound **4c**

**Figure S20.** ^11^B NMR of compound **4c**

*Catalyst preparation*: a mixture of CuBr (6 mg, 0.042 mmol) and hexabenzyltren (34 mg, 0.049 mmol) in dry degassed DCM (8 mL) was stirred for 6 hours at room temperature.

*Synthesis of compound* ***T1***

Compound **4a** (30 mg, 0.016 mmol) was placed in a dry flask under argon containing dry DCM (10 mL). Then, TBAF solution 1.0 M in THF (6 μL, 0.006 mmol) was added dropwise. The mixture was stirred for 18 hrs. The solvent was evaporated and the crude mixture was purified by column chromatography on silica gel (DCM/MeOH 100/7) to give the deprotected derivative in 50% yield. This derivative (14 mg, 0.008 mmol) was placed in a dry flask under argon containing dry DCM (10 mL). Then, compound **5** (7 mg, 0.009 mmol), and the prepared catalyst solution (4 mL) was added and the reaction mixture was stirred at room temperature for 48 hours with exclusion of light. Then, the solution was washed twice with 20 mL of an aqueous solution of EDTA (0.05 M), then with 20 mL of water. The organic phase was dried over anhydrous MgSO_4_, then filtered, and finally concentrated under reduced pressure. After purification by column chromatography on silica gel (DCM/MeOH 100/7) a blue solid was obtained in 53 % yield. **^1^H NMR (300 MHz, C_5_D_5_N)** δ: 1.63 (s, 6H, H_D_), 1.66-1.93 (m, 34H, CH_3_Ala, CH_3_Aib, H_10’_ H_7_, H_3_, H_6_, H_8_), 2.02-2.49 (m, 9H, H_j_, H_2_, H_5_, H_d_, H_10’’_), 2.92 (s, 6H, H_A_), 3.02-3.18 (m, 6H, H_c_, H_k_, H_11_), 3.34-3.74 (m, 4H, H_4,_ H_9_), 4.29-4.39 (m, 13H, H_Fc_), 4.40-4.54 (m, 4 H, CHAla, H_1_), 4.55-4.69 (m, 3H, CHAla, H_H_), 4.69-4.74 (m, 5H, H_Fc_), 4.74-4.88 (m, 4H, H_e_, H_i_), 4.88-5.44 (m, 4H, CHAla, H_G_), 7.72-7.82 (m, 4H; H_arom_), 7.89-8.24 (m, 5H, NH, H_a_, and H_m_), 8.53-8.63 (m, 1H, NH), 9.24-9.36 (m, 1H, NH), 9.45-9.60 (s, 1H, NH); **^11^B NMR (96 MHz, C_5_D_5_N)** δ: 3.60 (t, J= 34.5 Hz); **HRMS (ESI+)** m/z calcd for C_150_H_108_BF_2_Fe_2_N_15_O_12_S_2_: 2537.6631 [M+H]^+^, found 2537.6829.

**Figure S21.** ^1^H NMR spectra in C_5_D_5_N (300 MHz, 298K) of the target assembly **T1**. The peaks labelled with an asterisk (* and #) are due to ligand residues and water, respectively.

**Figure S22.** ^1^H NMR spectra of **T1** (enlargement of the 6.5-10 ppm region). The peaks labelled with an asterisk * are due to ligand residues.

**Figure S23.** ^1^H NMR spectra of **T1** (enlargement of the 6.5-10 ppm region). The peaks labelled with an asterisk (* and #) are due to ligand residues and water, respectively.

**Figure S24.** ^11^B NMR of compound **T1**

**Figure S25.** Mass spectrum of compound **T1**.

**Figure S26.** Theoretical and experimental [M+H]^+^ masses of compound **T1**.

*Synthesis of compound* ***T2***

Compound **4b** (45 mg, 0.0238 mmol) was placed in a dry flask under argon containing dry DCM (5 mL). Then, TBAF solution 1.0 M in THF (7 μL, 0.007 mmol) was added dropwise. The mixture was stirred overnight. The solvent was evaporated and the deprotected derivative was placed in a dry flask under argon containing dry DCM (10 mL). Then, compound **5** (21 mg, 0.0285 mmol), and the prepared catalyst solution (4 mL) was added and the reaction mixture was stirred at room temperature for 48 hours with exclusion of light. Then, the solution was washed twice with 20 mL of an aqueous solution of EDTA (0.05 M), then with 20 mL of water. The organic phase was dried over anhydrous MgSO_4_, then filtered, and finally concentrated under reduced pressure. After purification by column chromatography on silica gel (DCM/MeOH 100/7) a blue solid was obtained in 72 % yield. **^1^H NMR (300 MHz, C_5_D_5_N)** δ: 1.11 (t, *J = 7.4 Hz*, 6H; H_B_), 1.44 (s, 6H, H_D_), 1.46-1.95 (m, 33H, CH_3_Ala, CH_3_Aib, H_10’_ H_7_, H_3_, H_6_, H_8_), 2.02-2.70 (m, 13H, H_C_, H_j_, H_2_, H_5_, H_d_, H_10’’_), 2.97-3.20 (m, 6H, H_11_, H_c_, H_k_), 3.31-3.77 (m, 3H, H_4,_ H_9_), 4.30-4.38 (m, 9H, H_Fc_), 4.40-4.55 (m, 8H, CHAla, H_1_, H_Fc_), 4.56-4.72 (m, 3H, CHAla, H_H_), 4.71-4.89 (m, 7H, H_e_, H_i_, H_Fc_), 4.91-5.06 (m, 4H, CHAla, H_G_), 7.24-7.39 (m, 4H; H_arom_), 7.50 (d, *J = 16.5 Hz*, 2H, H_A_), 7.79 (t, *J= 5.2 Hz*, 1H, NH), 7.91-8.22 (m, 7H, NH, H_a_, H_m_, H_A_), 8.61 (d, *J= 6.0 Hz*, 1H, NH), 9.29 (d, *J= 3.2 Hz,* 1H, NH), 9.50 (s, 1H, NH); **^19^F NMR (282 MHz, C_5_D_5_N**) δ: -137.11 (q); **^11^B NMR (96 MHz, C_5_D_5_N)** δ: 3.38 (t). **HRMS (ESI+)** m/z calcd for C_152_H_116_BF_2_Fe_2_N_15_O_12_S_2_: 1284.8628 [M^+•^ + H]^2+^, found 1284.8578.

**Figure S27.** ^1^H NMR spectra in C_5_D_5_N (300 MHz, 298K) of the target assembly **T2**.

**Figure S28.** ^1^H NMR spectra of **T2** (enlargement of the 6.5-10 ppm region).

**Figure S29.** ^1^H NMR spectra of **T2** (enlargement of the 0-5.5 ppm region).

**Figure S30.** ^19^F NMR spectra of **T2.**

**Figure S31.** ^11^B NMR spectra of **T2.**

**Figure S32.** Mass spectrum of compound **T2**.

**Figure S33.** Mass difference report on [M^+•^ + H]^2+^ masses of compound **T2**.

*Synthesis of compound* ***T3***

Compound **4c** (35 mg, 0.017 mmol) was placed in a dry flask under argon containing dry DCM (6 mL). Then, TBAF solution 1.0 M in THF (5 μL, 0.005 mmol) was added dropwise. The mixture was stirred overnight. The solvent was evaporated and the deprotected derivative was placed in a dry flask under argon containing dry DCM (10 mL). Then, compound **5** (15 mg, 0.020 mmol), and the prepared catalyst solution (3 mL) was added and the reaction mixture was stirred at room temperature for 9 hours with exclusion of light. Then, the solution was washed twice with 20 mL of an aqueous solution of EDTA (0.05 M), then with 20 mL of water. The organic phase was dried over anhydrous MgSO_4_, then filtered, and finally concentrated under reduced pressure. After purification by column chromatography on silica gel (DCM/MeOH 8/0.5) a green solid was obtained in 70 % yield. **^1^H NMR (300 MHz, C_5_D_5_N)** δ: 1.11 (t, *J = 7.4 Hz*, 6H; H_B_), 1.44 (s, 6H, H_D_), 1.46-1.95 (m, 33H, CH_3_Ala, CH_3_Aib, H_10’_ H_7_, H_3_, H_6_, H_8_), 2.02-2.70 (m, 13H, H_C_, H_j_, H_2_, H_5_, H_d_, H_10’’_), 2.97-3.20 (m, 6H, H_11_, H_c_, H_k_), 3.31-3.77 (m, 3H, H_4,_ H_9_), 4.30-4.38 (m, 14H, H_Fc_), 4.40-4.55 (m, 3H, CHAla, H_1_,), 4.61-4.76 (m, 6H, H_Fc_, H_H_), 4.78-4.89 (m, 4H, H_e_, H_i_), 4.91-5.06 (m, 5H, CHAla, H_G_), 7.24-7.39 (m, 4H; H_arom_), 7.50 (d, *J = 16.7 Hz*, 2H, H_A_), 7.63 (d, *J= 8.3 Hz*, 4H), 7.75 (d, *J=8.3 Hz*, 4H), 7.99 (s, 1H, Hm), 8.06 (d, *J= 5.3 Hz*, 1H, NH), 8.10 (s, 1H, NH), 8.16-8.18 (s, d, 2H, Ha, NH), 8.39 (d, *J=16.7 Hz*, 2H, H_A_), 8.61 (d, *J= 6.0 Hz*, 1H, NH), 9.29 (d, *J= 3.3 Hz*, 1H, NH), 9.50 (s, 1H, NH); **^19^F NMR (282 MHz, C_5_D_5_N**) δ: -135.51(m); **^11^B NMR (96 MHz, C_5_D_5_N)** δ: 4.47 (t). **MS (ESI+)** m/z calcd for C_168_H_124_BF_2_Fe_2_N_15_O_12_S_2_Na: 2791.7705 [M+Na]^+^, found 2791.7043.

**Figure S34.** ^1^H NMR spectra in C_5_D_5_N (300 MHz, 298K) of the target assembly **T3**.

**Figure S35.** ^1^H NMR spectra of **T3** (enlargement of the 6.5-10 ppm region).

**Figure S36.** ^1^H NMR spectra of **T3** (enlargement of the 0.6-5.2 ppm region).

**Figure S37.** ^19^F NMR spectra of **T3.**

**Figure S38.** ^11^B NMR spectra of **T3.**

**Figure S39.** Mass spectrum of compound **T3**.

**Figure S40.** Theoretical and experimental [M+Na]^+^ masses of compound **T3**.

**Figure S41.** Normalized absorption spectra of (Fc)_2_-BDP **1a** and BODIPY model compound **1d**.

**Figure S42.** Normalized absorption spectra of (Fc)_2_-BDP **1b** and DSBDP.

**Figure S43.** Normalized absorption spectra of (Fc)_2_-BDP **1c** and BODIPY model compound **1e**.

**Figure S44.** Normalized absorption spectra of (Fc)_2_-BDP **1a**-**1c** and fullerene platform **3**.

**Figure S45.** Normalized absorption spectra of (Fc)_2_-BDP-C_60_ conjugates **4a**-**4c** and the molecular assemblies (Fc)_2_-BDP-C_60_-peptide **T1**-**T3**. Inset: Absorption spectrum of hexapeptide **5**.

**Figure S46.** Emission spectra of compounds **1b**-**1c** and **4b**-**4c**, recorded in DCM. Top: Reference molecules **BDP** (λexc=495 nm) and **DSBDP** (λexc=605 nm) and (Fc)_2_-BDP precursors **1b** (λexc=550 nm) and **1c** (λexc=590 nm). Bottom: **DSBDP** (λexc=605 nm) and (Fc)_2_-BDP-C_60_ target assemblies **4b** (λexc=550 nm) and **4c** (λexc=605 nm).

**Table S1.** Absorption and fluorescence data of the triads **1a-1c** and tetrads **4a-4c**, recorded in DCM.

| **Compound** | **λ_BDP_^abs^**  **(nm)** | **ε**  **(L.mol^-1^.cm^-1^)** | **λ_max_ ^em^**  **(nm)** | **Φ**  **(% ±5 %)** |
| --- | --- | --- | --- | --- |
| **1a** | 579 | 29000 | 545 | 0.1[^a^] |
| **1b** | 692 | 37000 | 594 | 0.2[^b^] |
| **1c** | 662 | 60100 | 680 | 0.1[^c^] |
| **4a** | 579 | 30200 | 540 | 0.08^[a]^ |
| **4b** | 693 | 46700 | 595 | < 0.01^[b]^ |
| **4c** | 670 | 44100 | 674 | < 0.02^[c]^ |

^[a]^ Rhodamine 6G used as standard (φ = 0.95 in ethanol), ^[b]^ Sulforhodamine 101 used as standard (φ = 0.90 in ethanol), ^[c]^ Oxazine 1 used as standard (φ = 0.11 in ethanol).

**Figure S47.** Cyclic voltammograms of the triad **1a-1c** in anhydrous and oxygen-free DCM, 0.1M of *n*-Bu_4_NPF_6_, pseudo-reference electrode: Ag wire, working electrode: Pt, auxiliary electrode: Pt, scan rate: 100 mV.s^-1^.

**Figure S48. left)** Scan-rate dependence of the cyclic voltammograms; **middle)** peak current as a function of scan rate; **right)** Experimental (dots) and simulated (lines) peak to peak potential variation versus scan rates of the target assemblies SAMs on gold microelectrodes. Deviations from simulations at larger scan rates results from instrumental distortions by the potentiostat (bandwidth limitation).

**Table S2.** Molecular coverages Γ and capacitances per surface areas C.

| **Compound** | **Γ (mol.cm^-2^)×10^11^** | **C (µFcm^-2^)** |
| --- | --- | --- |
| **T1** | 2.5 | 5 |
| **T2** | 3.4 | 3 |
| **T3** | 5.2 | 4 |

**Figure S49.** Estimated molecular footprint of (Fc)_2_-BDP-C_60_-peptides assemblies **T1**, **T2** and **T3** assuming that these structures are in extended conformations and that the hexapeptide adopts an helical conformation.

1. [] a) H. Nasrallah, J. Rabah, V. Bui-Thi-Tuyet, K. Baczko, H. Fensterbank, F. Bourdreux, A. M. Goncalves, V. Declerck, S. Boujday, V. Humblot, K. Wright, A. Vallée and E. Allard, *New Journal of Chemistry* **2018**, *42*, 19423-19432; b) J. Rabah, L. Yonkeu, K. Wright, A. Vallée, R. Méallet-Renault, M.-H. Ha-Thi, A. Fatima, G. Clavier, H. Fensterbank and E. Allard, *Tetrahedron* **2021**, *100*, 132467; c) J. Rabah, A. Fatima, H. Fensterbank, K. Wright, A. Vallée, M. Gueye, G. Burdzinski, G. Clavier, F. Miomandre, J. Pham, M. Sliwa, R. Méallet‐Renault, K. Steenkeste, T. Pino, M. Ha‐Thi, E. Allard, *Helv. Chim. Acta* **2023**, *106*, e202300039; d) J. Pham, J.-F. Audibert, J. Rabah, K. Wright, J. Marrôt, M.-H. Ha-Thi, R. Méallet-Renault, E. Allard, F. Miomandre, *J. Phys. Chem. C* **2024**, *128*, 15162. e) Jad Rabah. BODIPY-[60]Fullerene–based donor-acceptor systems for organic electronics : Synthesis, Characterization, and Surface Modification. Université Paris-Saclay, **2020**, https://theses.hal.science/tel-03225361
2. [] L. Liang, J. Ruiz and D. Astruc, *Advanced Synthesis & Catalysis* **2011**, *353*, 3434.
3. [] A. Vallée, V. Humblot, R. Al Housseiny, S. Boujday and C.-M. Pradier, *Colloids and Surfaces B: Biointerfaces* **2013**, *109*, 136.
4. [] Y. Miura, S. Kimura, Y. Imanishi and J. Umemura, *Langmuir* **1998**, *14*, 6935.
5. [] K. Yanagisawa, T. Morita and S. Kimura, *Journal of the American Chemical Society* **2004**, *126*, 12780.
6. [] M. Tsubio, *Journal of Polymer Science* **1962**, *59*, 139.
7. [] C. Amatore, E. Maisonhaute, B. Schöllhorn and J. Wadhawan, *ChemPhysChem* **2007**, *8*, 1321.
8. [] A. J. Bard and L. R. Faulkner, *Electrochemical Methods: Fundamentals and Applications*, Wiley India Ltd.: New Delhi, **2004**, p.
9. [] Y. Wang, E. I. Rogers and R. G. Compton, *Journal of Electroanalytical Chemistry* **2010**, *648*, 15.
10. [] a) P. Fortgang, M. Urbani, M. Holler, J. F. Nierengarten, A. Moreau, B. Delavaux-Nicot and E. Maisonhaute, *Electroanalysis* **2015**, *27*, 1010; b) L. Le-Quang, R. Farran, Y. Lattach, H. Bonnet, H. Jamet, L. Guérente, E. Maisonhaute and J. Chauvin, *Langmuir* **2018**, *34*, 5193.
